# The Influence of Hydroxylation on Maintaining CpG Methylation Patterns: A Hidden Markov Model Approach

**DOI:** 10.1371/journal.pcbi.1004905

**Published:** 2016-05-25

**Authors:** Pascal Giehr, Charalampos Kyriakopoulos, Gabriella Ficz, Verena Wolf, Jörn Walter

**Affiliations:** 1 Department of Biological Sciences, UdS, Saarbrücken, Saarland, Germany; 2 Computer Science Department, UdS, Saarbrücken, Saarland, Germany; 3 Barts Cancer Institute, Queen Mary University, London, United Kingdom; Friedrich Miescher Institute for Biomedical Research, SWITZERLAND

## Abstract

DNA methylation and demethylation are opposing processes that when in balance create stable patterns of epigenetic memory. The control of DNA methylation pattern formation by replication dependent and independent demethylation processes has been suggested to be influenced by Tet mediated oxidation of 5mC. Several alternative mechanisms have been proposed suggesting that 5hmC influences either replication dependent maintenance of DNA methylation or replication independent processes of active demethylation. Using high resolution hairpin oxidative bisulfite sequencing data, we precisely determine the amount of 5mC and 5hmC and model the contribution of 5hmC to processes of demethylation in mouse ESCs. We develop an extended hidden Markov model capable of accurately describing the regional contribution of 5hmC to demethylation dynamics. Our analysis shows that 5hmC has a strong impact on replication dependent demethylation, mainly by impairing methylation maintenance.

## Author Summary

Oxidation of 5mC by Ten-eleven translocation (Tet) enzymes leads to the formation of 5hmC and other higher oxidized forms in the DNA. Several findings indicate that oxidation induces demethylation processes, but the mechanistic contribution of 5hmC to this process remains unclear. Using an innovative combination of 5hmC detection chemistry and high resolution sequencing, we generate data that can be used for a novel hidden Markov modeling approach. This new model for the first time incorporates 5hmC dynamics and allows to test certain scenarios of demethylation mechanisms. Our findings support the conclusion that 5mC oxidation compromises the copying of DNA methylation patterns across generations in ES-cells.

## Introduction

DNA methylation is an epigenetic modification essential for the regulation of genome stability and genome function [1, 2]. During development the distribution of DNA methylation is under strict control to maintain a temporal and cell type specific persistence of epigenetic information [3]. The methylation of DNA in mammals is restricted to the C-5 position of cytosine and is predominantly found in a CpG sequence context [4, 5].

**Fig 1:**
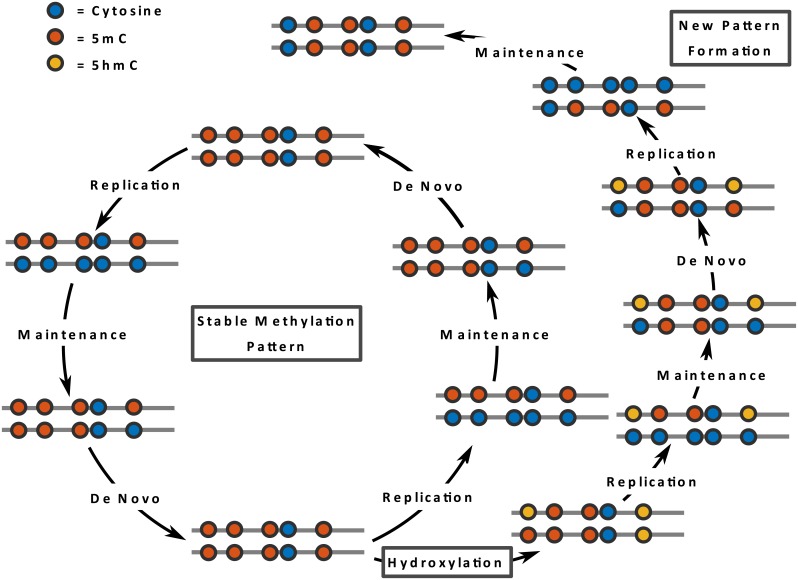
Maintenance and de novo methylation are usually cooperating to maintain a stable methylation pattern (inner circle). The oxidation of 5mC to 5hmC may interfere with the maintenance machinery causing a (partial) loss of CpG methylation after DNA replication. DNA strands are indicated by lines whereas the CpG are shown as colored circles.

Our current knowledge suggests that DNA methylation patterns (5mC) are mainly established by DNA methyltransferases Dnmt3a and Dnmt3b [3, 6]. The palindromic nature of a CpG sequence in which 5mC occurs allows a recognition of the 5mC hemimethylated state after semi-conservative replication and a copying of the parental methylation pattern to the newly synthesized DNA strand (see Fig 1). A series of experiments revealed that Dnmt1 in conjunction with Uhrf1 are responsible for this copying also referred to as *maintenance* methylation. Dnmt1 and Uhrf1 have a high preference for binding to hemimethylated CpG substrates [7–9]. Together they assure the maintenance symmetric CpG methylation patterns after each round of replication.

In contrast to Dnmt1, Dnmt3a and Dnmt3b act on hemi- as well as unmethylated CpGs and their activity is not coupled to DNA replication. Both enzymes are highly regulated and regarded as the main enzymes to establish new methylation patterns and are therefore classified as *de novo* DNA methyltransferases. However, recent data shows that Dnmt1 may also de novo methylate unmethylated dyads and that Dnmt3a and Dnmt3b are also involved in reestablishing (thus “maintaining”) complete methylation patterns at certain loci [10]. In summary, the persistence of methylation patterns is controlled by a coordinated action of de novo and maintenance functions of all three enzymes.

Besides the establishment and the persistence of methylation its removal is also of great biological importance. Demethylation events can occur on a local scale in case of individual gene activation but also on a global genome wide level like in the early zygote and the germ line, where genomes are reprogrammed for new developmental functions [11, 12]. In both cases demethylation can be achieved either by an active mechanism (direct removal), a passive replication-dependent loss or a combination of both.

Recent findings suggest that the oxidation of 5mC modulates active and passive demethylation processes. 5-hydroxymethyl cytosine (5hmC) is generated by oxidation of 5mC in an enzymatic reaction catalyzed by the oxoglutarate- and Fe(ii)-dependent ten-eleven trans-location dioxygenases (Tet1, Tet2, and Tet3) [13]. Tet enzymes also catalyze further oxidations to 5-formylcytosine (5fC) and to 5-carboxycytosine (5caC), which have been shown to promote processes of active demethylation [14–16]. Still 5hmC is the most prevalent oxidation type and widely discussed to having an influence on DNA methylation pattern stability in dividing cells. 5hmC not only alters the chemical properties but also the biological recognition of the base. Dnmt1 binds to 5hmC with a much lower efficiency than to 5mC. This may impair the replication dependent copying of 5mC [17].

In mouse ES cells (mESCs), in the early mouse embryo and in the early germ cells DNA demethylation stability is influenced by the conversion of 5mC into 5hmC. Disturbances or depletion of Tet enzymes in these phases result in massive changes of 5hmC and lead to developmental consequences [18–20]. These findings indicate that the controlled alteration of DNA methylation patterns across DNA replications is dependent on 5hmC. However, the underlying mechanisms are still under debate. Mouse ESCs are a well established system to study these effects as they rapidly lose DNA methylation on a genome wide scale when the cells are transferred from conventional serum medium containing LIF (primed state) to a synthetic 2i medium [21, 22]. This loss of 5mC is coupled to a temporary gain of 5hmC. In our study we follow the dynamic of DNA demethylation in mESCs over time and DNA replications using a novel combination of hairpin sequencing with bisulfite sequencing (BS) and oxidative bisulfite sequencing (oxBS). This method allows us to determine the methylation status of both complementary DNA strands at individual chromosomes and the status of 5hmC levels at given time points [10, 23, 24].

We propose a stochastic model that describes the evolution of both methylation and hydroxylation patterns over time. Our model allows that methylation can be lost due to cell replication and methyl groups can be added due to either maintenance or de novo enzyme activity [10, 25]. In addition, we assume that all methylated sites can be hydroxylated.

Based on these assumptions we define a hidden Markov model (HMM) for each data set and construct likelihood functions on the basis of the two sequencing methods. The combination of the two likelihoods allows us to derive estimations for the levels of (hydroxy-)methylation based on observations at four different time points. Finally, we determine unknown parameters of the model, i.e., methylation and hydroxylation efficiencies as well as the initial distribution of the hidden states. Despite its simplicity, the model accurately predicts the evolution of the (hydroxy-)methylation patterns and allows us to test different assumptions about the activities of the involved enzymes.

## Methods

### Hairpin oxidative bisulfite sequencing

**Fig 2:**
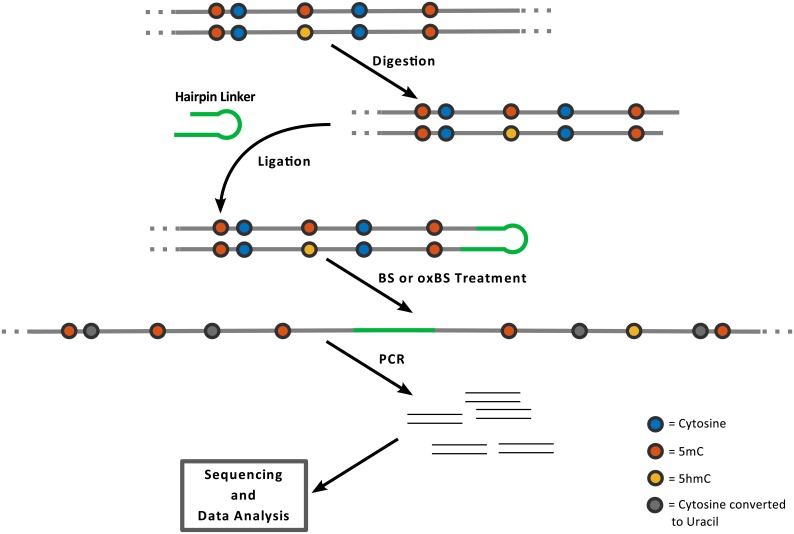
Schematic outline of hairpin bisulfite (BS) and oxidative bisulfite sequencing (oxBS) methods: The method is based on enzymatic digestion of genomic DNA and the covalent connection of upper and lower DNA strands by ligating a hairpin oligonucleotide. PCR enrichment of the BS/oxBS treated sample is used for amplicon generation followed by sequencing and data analysis.

Currently no comprehensive data are available allowing to model the fate of 5hmC at a single base resolution level. Therefore, extending the method described in Fitz et al. 2014 and Arand et al. [10, 21] we established a workflow enabling us to produce such data. To obtain base resolution information of the modification status we apply hairpin bisulfite sequencing on DNA samples split into oxidative (oxBS-Seq) and non oxidative standard bisulfite reaction (BS-Seq) data sets. The use of the hairpin linker strategy allows us then to determine the levels of 5hmC and 5mC on both DNA strands [23] and to determine the methylation status (hemimethylated, unmethylated or fully methylated) at each individual CpG dyad within the sequenced loci at single molecule resolution. To obtain a sufficient coverage (>1000x) per CpG we use very deep NGS based sequencing of selected loci. The deep sequencing enables us to determine accurate rates and error rates for each modification. To cover larger parts in the genome we included the analysis of mobile elements which occur in multiple identical copies across the genome and to which we refer as “repetitive elements”. In fact our analysis covers about 91% of all annotated IAP(IAPLTR1a_mM) (N = 1635), 20% of L1md_A (N = 3287), 12% for L1md_T (N = 2784) and 30% of MuERVL (N = 725). In this case the >1000x coverage has to be seen as the aggregate of about a 5x coverage of each individual copy of a given repetitive element. Fig 2 outlines the main experimental steps of the procedure.

In the first step genomic DNA is digested using restriction enzymes which generate cuts close to the gene/locus selected for methylation analysis. In a following reaction both DNA strands are ligated to a back-folding “hairpin”-oligonucleotide. Next the DNA is unfolded and subjected to a bisulfite or oxidative bisulfite treatment followed by a locus specific PCR amplification. PCR primers contain Mi-Seq (Illumina) compatible extensions to perform deep (paired end 2x300bp) sequencing (up to 10K/product). Sequencing data are processed using our in house software BiQ-HT and a python script. In the bisulfite only reaction 5mC and 5hmC remain as cytosines, while in the oxidative bisulfite reaction 5hmC is converted to uracil/thymine. Each individual sequence covers the hairpin linker which contains modified and unmodified cytosines at known positions. This allows us to monitor the efficacy of bisulfite and oxidative bisulfite reactions per molecule (note that all unmodified cytosines are converted to thymines) and calculate exact error rates by dividing the number of unconverted bases by the total number of analyzed cytosines. Additional information about the protocol is given in S1 Text together with reference-, primer- and linker-sequences.

### Hidden Markov model

Our model considers a CpG site (alternatively dyad) over time and describes its state as a (discrete time) Markov chain {X(t),t∈N} taking values in $S={\left\{u,m,h\right\}}^{2}$. Each state (*s*_1_, *s*_2_) (for *s*_1_, *s*_2_ ∈ {*u*, *m*, *h*}) encodes whether the upper strand (lower strand) is *unmethylated* (*u*), *methylated* (*m*) or *hydroxylated* (*h*). For instance, in state (*s*_1_, *s*_2_) = (*u*, *h*) the upper strand is unmethylated and the lower strand is hydroxylated. We will often simply write (*s*_1_ *s*_2_) instead of (*s*_1_, *s*_2_).

The time parameter *t* corresponds to the number of cell divisions and the state transitions are triggered by three consecutive events: cell division, methylation and hydroxylation. The corresponding transition probability matrices are **D**(*t*), **M**(*t*), and **H**(*t*), respectively. Thus, the combined transition probability matrix of X is defined as

$$\begin{array}{c} P(t)=D(t)\cdot M(t)\cdot H(t), \end{array}$$

with entries **P**_*ij*_(*t*) that equal the probabilities that given $X\left(t\right)=i=\left(s_{1}s_{2}\right)$, the next state is $X\left(t+1\right)=j=\left(s_{1}^{\prime }s_{2}^{\prime }\right)$ for all i,j∈S. Note here we assume that hydroxylation occurs after methylation to ensure that between two cell divisions a transition from *u* to *m* and then to *h* is possible. Moreover, note that we allow **P**(*t*) to change over time, so that we capture the case that the (hydroxy-)methylation efficiencies do not remain constant over time. In the sequel we give a detailed description of **D**(*t*), **M**(*t*), and **H**(*t*). For a formal definition of the matrices, we refer to S1 Text.

#### Demethylation through cell division

**Fig 3:**
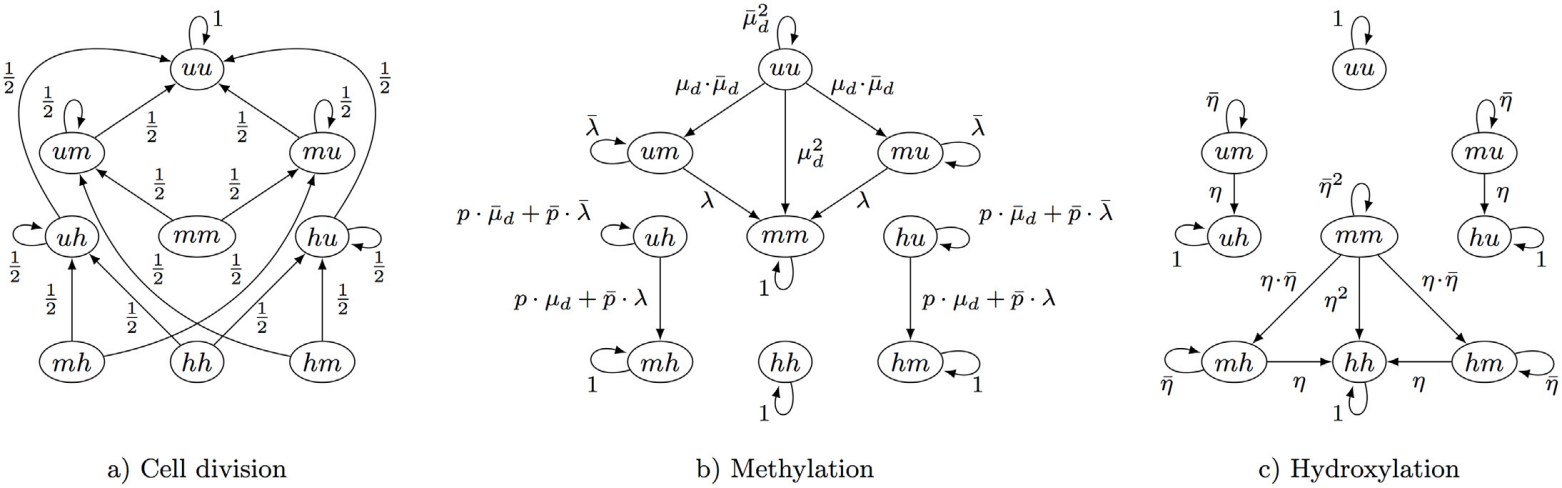
Possible transitions of the 9 different states of a CpG site. Methyl groups are a) removed after cell division, b) added due to maintenance (*μ*_*m*_) or de novo methylation (*μ*_*d*_) and c) are hydroxylated (*η*) by Tet enzymes.

With each cell division and DNA replication one new DNA strand is synthesized resulting in a temporary situation where only unmodified cytosines are present in the new strand. Since the epigenetic pattern of the parental strand remains unchanged a previously methylated CpG site keeps half of the (hydroxy-)methylated state in the two daughter cells. By averaging over the daughter cells, if the current state is (*mm*) then after cell division the new state is (*um*) or (*mu*) each with probability 0.5 (depending on whether the newly synthesized strand is the upper or the lower strand). Similarly, with probability 0.5 the process enters (*uh*) or (*hu*) from (*hh*). Thus, DNA replication/cell division may result in a direct loss of methyl or hydroxyl groups. The transition probabilities of the remaining states are defined in a similar way and we illustrate the corresponding matrix **D**(*t*) in Fig 3a).

#### Methylation

The loss of methylation by DNA replication is counteracted by a restored methylation due to the combined activity of the three methyltransferases Dnmt1, Dnmt3a and Dnmt3b. We distinguish between maintenance methylation catalyzed by Dnmt1 and de novo methylation catalyzed by Dnmt3a and Dnmt3b. We assume that a cytosine of an unmethylated dyad can only be methylated by a de novo event, while both maintenance and de novo methylation are possible on a hemimethylated dyad. Based on related in vitro experiments [3] and our recently published work [10], we assume that Dnmt3a/b act on hemimethylated sites with the same efficiency as on unmethylated sites.

We define *μ*_*m*_(*t*) and *μ*_*d*_(*t*) as the probabilities of maintenance and de novo methylation of a cytosine, respectively, where the corresponding methylation event occurs within the *t*-th cell division cycle (*t* ∈ {1, 2, …}). In addition, we define λ(*t*) to be the total methylation efficiency on a hemimethylated site. It holds that

$$\begin{array}{c} \lambda \left(t\right)=\mu_{m}\left(t\right)+\mu_{d}\left(t\right)-\mu_{m}\left(t\right)\cdot \mu_{d}\left(t\right), \end{array}$$

because maintenance is associated with the replication machinery and happens immediately after replication with efficiency *μ*_*m*_(*t*). In case maintenance methylation by Dnmt1 is not successful the site can still be methylated with de novo methylation efficiency *μ*_*d*_(*t*) which then gives λ(*t*) = *μ*_*m*_(*t*) + (1 − *μ*_*m*_(*t*)) ⋅ *μ*_*d*_(*t*). We write ${\bar{\mu }}_{m}\left(t\right)=1-\mu_{m}\left(t\right),$ ${\bar{\mu }}_{d}\left(t\right)=1-\mu_{d}\left(t\right)$ and $\bar{\lambda }\left(t\right)=1-\lambda \left(t\right)$ for the complements of the above probabilities and we omit the time parameter *t* whenever it is not relevant.

Note that if a CpG site has two unmethylated cytosines then two de novo methylation events are possible. Assuming independence between them, all transition probabilities of the corresponding state (*uu*) are the product of two event probabilities. We illustrate the corresponding methylation matrix **M**(*t*) in Fig 3b). Here *p* is the probability that maintenance methylation is not applied to the states (*hu*) and (*uh*), i.e., the hydroxyl group prevents the maintenance process, i.e., the methylation of the unmodified cytosine on the opposite strand. As a result, from these states the states (*hm*) and (*mh*) can only be entered via de novo methylation. In the opposite case, with probability $\bar{p}=1-p$, states (*hu*) and (*uh*) are seen as hemimethylated during maintenance and can enter states (*hm*) and (*mh*) with probability λ for both maintenance and de novo methylation (see Fig 3b). Besides, the states (*mh*), (*hm*), and (*hh*) have only self-loops since the Dnmts do not modify hydroxyl groups.

#### Hydroxylation

Let *η*(*t*) be the probability that before the (*t* + 1)-th cell division a methylated position becomes hydroxylated, i.e, the probability of a transition from *m* to *h*. Similarly as above, we write $\bar{\eta }\left(t\right)$ for 1 − *η*(*t*) and omit *t* whenever convenient. Assuming again independence between two hydroxylation events, the corresponding matrix **H**(*t*) is illustrated in Fig 3c). Note that without an active hydroxylation mechanism (*η* > 0) the level of 5hmC would half after each replication since newly synthesized strands do not inherit the hydroxyl groups of the mother strand.

Hydroxylation is the last modification that we consider before the next cell division. Thus, between two cell divisions an unmethylated position may transition from *u* to *m* and then to *h*.

#### Observable states and conversion errors

**Fig 4:**
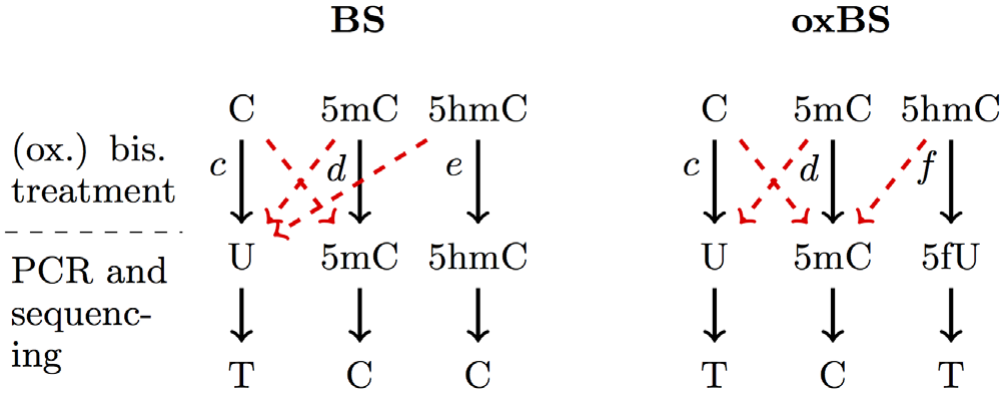
Schematic outline of the conversion of Cytosine, 5mC and 5hmC during BS and oxBS treatment and after sequencing: In the bisulfite reaction a cytosine (C) is converted to uracil (U), whereas 5mC and 5hmC remain untouched. In the oxidative bisulfite sequencing only 5mC remains untouched and cytosine as well as 5hmC is converted to uracil (U). The conversion errors are illustrated as dashed red arrows and *c*, *d*, *e*, *f* are the conversion probabilities.

In order to define the observable states and the corresponding emission probabilities, we first describe the details of hairpin sequencing and (oxidative) bisulfite sequencing. First the DNA is cut by a restriction enzyme. The DNA fragments are then linked covalent to a Hairpin linker resulting in the connection of upper and lower strand. The resulting hairpin fragments are divided into two halves, one is treated with a standard bisulfite reaction and the other is subjected to an oxidation followed by bisulfite treatment. Both 5mC and 5hmC are not affected by the (non-oxidative) bisulfite treatment and appear after sequencing as cytosines. In the oxidative case 5hmC is oxidized to 5fC which is converted during bisulfite treatment to 5fU and represents itself after sequencing as thymine (see Fig 4).

We incorporated unmodified cytosine as well as 5mC and 5hmC into the hairpin linker to precisely estimate the conversion errors (see also S1 Text) that influence the transition probabilities between the hidden and the observable states. These controls allow us to correct for technical errors in individual measurements.

In Fig 4 the transitions from a site’s possible hidden states to the observable ones are shown. Each base will eventually transform into a thymine (T) or a cytosine (C). Thus, the set of the observable states for a CpG site with two cytosines is $S_{obs}={\left\{T,C\right\}}^{2}.$ The red dashed arrows correspond to conversion errors and assuming all errors are zero, i.e., the probabilities *c* = *d* = *e* = *f* of a correct conversion are all one, a C will eventually transform to T and a 5mC will transform to C in both bisulfite and oxidative bisulfite setups. However, a hydroxylated cytosine (5hmC) is ideally mapped to a C during BS and to a T during oxBS. The entries of the corresponding emission matrices **E**_*bs*_(*t*) and **E**_*ox*_(*t*) for the transitions from hidden to all observable states can be found in Table A in S1 Text and the values of the conversion errors from all analyzed loci for each of the experimental setups are listed in S1 and S2 Tables. Note that the values of *c* and *d* can differ between the two treatments and that the conversion probabilities can also differ over time.

#### Estimation of model parameters

Given the number of times *n*_*bs*_(*j*, *t*) and *n*_*ox*_(*j*, *t*) that state $j\in S_{obs}={\left\{T,C\right\}}^{2}$ has been observed during independent BS and oxidative BS measurements at time *t* we use a maximum likelihood approach to estimate the unknown parameters of the HMMs, that is, the initial distribution of the hidden states, $S={\left\{u,m,h\right\}}^{2}$, the unknown functions *μ*_*d*_(*t*), *μ*_*m*_(*t*) and *η*(*t*), as well as the probability *p* at which CpG sites with one hydroxyl group are not considered during maintenance.

Formally, let *π*(*t*) be the row vector of the state probabilities of the hidden states after *t* cell divisions, i.e., *π*(0) is the initial distribution of the hidden states. For i∈S let π(i,t)=P(X(t)=i) denote the entry of *π*(*t*) that corresponds to state *i*. The probability of observing state $j\in S_{obs}$ at time *t* is given by

$$\begin{array}{c} P\left(O\left(t\right)=j\right)=\sum_{i\in S}P\left(O\left(t\right)=j|X\left(t\right)=i\right)\cdot \pi \left(i,t\right), \end{array}$$

where O(t) is the random variable for the state observed at time *t* and P(O(t)=j∣X(t)=i) is the emission probability. In matrix-vector form this yields

$$\begin{array}{c} \pi_{bs}\left(t\right)=\pi \left(t\right)\cdot E_{bs}\left(t\right)\;\;\text{and}\;\;\pi_{ox}\left(t\right)=\pi \left(t\right)\cdot E_{ox}\left(t\right) \end{array}$$

for the two sequencing experiments (BS and oxBS, respectively). Here, *π*_*bs*_(*t*) and *π*_*ox*_(*t*) are the vectors with the distribution over the observable states at time *t*. Note that both HMMs have the same distribution *π*(*t*) for the hidden states (as for both experiments the same cell population is used) but different emission probabilities and that *π*(*t*) is given by $\pi \left(t\right)=\pi \left(0\right)\cdot \prod_{k=1}^{t}P\left(k\right).$

First, we estimate the initial distribution *π*(0) based on the initial independent BS and oxidative BS measurements under conventional serum conditions by considering the combined likelihood

$$\begin{array}{c} L_{1}\left(\pi \left(0\right)\right)=\prod_{j\in S_{obs}}\pi_{bs}{\left(j,0\right)}^{n_{bs}\left(j,0\right)}\cdot \pi_{ox}{\left(j,0\right)}^{n_{ox}\left(j,0\right)}. \end{array}$$ (1)

The above likelihood depends only on the unknown vector *π*(0) and the emission matrices and allows us to determine the initial distribution of the hidden states. We maximize the likelihood subject to the constraint ∑_*i*_ *π*(*i*, 0) = 1, i.e., $\pi {\left(0\right)}^{*}={arg max}_{\pi (0)}L_{1}\left(\pi \left(0\right)\right),$ where *π*(0) ranges over all vectors that sum to one. Then, given an estimate for *π*(0), we compute for *t* ∈ {1, 2, …} the state probabilities *π*(*t*) of the hidden states and consider the common likelihood

$$\begin{array}{c} L_{2}\left(v\right)=\prod_{t\in T_{obs}\setminus \left\{0\right\}}\prod_{j}\pi_{bs}{\left(j,t\right)}^{n_{bs}\left(j,t\right)}\cdot \pi_{ox}{\left(j,t\right)}^{n_{ox}\left(j,t\right)} \end{array}$$ (2)

for the observations at all remaining observation time points *t* ∈ *T*_*obs*_. Note that here we assume that the cells divide every 24 hours, hence *t* ranges over all days at which measurements were made (see also S1 Text). In addition, we can assume independence between the observations because during the measurement only a small fraction of cells is taken out of a large pool and thus it is unlikely that we pick two cells with a common descendant.

The likelihood $L_{2}\left(v\right)$ depends on the matrices **P**(*t*) and thus on the unknown functions *μ*_*d*_(*t*), *μ*_*m*_(*t*), *η*(*t*) and the probability *p*. We assume that the enzymes’ efficiencies are linear in *t*, i.e., each function is of the form *β*_0_ + *β*_1_ ⋅ *t*, which yields a vector **v** of seven unknown parameters in total. For estimating **v** we use again a maximum likelihood approach, i.e., we determine $v^{*}={arg max}_{v}L_{2}\left(v\right),$ under the appropriate constraints (see S1 Text). The maximization of the likelihoods in Eqs (1) and (2) is a (global) optimization problem for which it is convenient to minimize the negative logarithm of the likelihood. Deriving expressions for the first and second derivatives of the log-likelihood is straightforward and yields fast convergence of the gradient descent optimization routine with multiple starting values. Due to the large number of samples we expect our maximum likelihood estimators (MLEs) to be approximately unbiased and normally distributed. Moreover, we can compute the observed Fisher information matrix (FIM) and thus derive confidence intervals for all parameters (for details see S1 Text).

## Results

Previous genome wide analyses showed a high or moderate decrease of DNA methylation in ESCs transferred from serum into 2i medium [21, 22]. Furthermore, it was shown that the oxidation of 5mC to 5hmC is likely to contribute to this DNA demethylation [21]. The goal of our work was to develop a model which describes the 5hmC dependent molecular mechanisms that cause this loss of DNA methylation upon consecutive rounds of replication. For the modeling we generated an ultra deep DNA methylation data set of selected loci in mouse ES cells (ESCs) collected at defined time points after cultivation in 2i.

For our analysis we chose five multicopy, repetitive elements, IAPs (intracisternal A particle), L1mdA and L1mdT (both Long interspersed nuclear elements), MuERVL (Murine endogenous retrovirus) and mSat (major satellite), as well as four single copy loci in the genes Afp, Snrpn, Ttc25 and Zim3. It was already known that some of these repetitive elements are subject to demethylation. Ttc25 and Zim3 where previously shown to exhibit a less pronounced loss of methylation in the absence of Tet1/Tet2 in 2i medium. [21]. Imprinted genes such as Snrpn were shown to be “resistant” to demethylation in 2i.

Deep locus specific DNA methylation profiles were generated from mESCs grown in conventional serum/LIF medium (day0) and after their transfer and cultivation into 2i medium for 24h (day1), 72h (day3) and 144h (day6), respectively. During this period the ESCs undergo a maximum of six cell divisions (as inferred from cell densities). For each time point and locus we performed consecutive bisulfite and oxidative hairpin bisulfite reactions using high coverage Mi-Seq sequencing (see Methods section). Following sequence processing (alignment, trimming, QC filtering) we obtained two data sets for each locus: one describing the combined 5mC+5hmC status (BS-Seq) and one describing the 5mC status alone (oxBs-Seq). The hairpin refolding of sequences then let us determine the accurate double stranded CpG methylation status at a given locus (hemi-, fully- or unmethylated).

With this data we used our HMMs (described in the Methods section) to estimate the amount of 5mC and 5hmC in these loci and to predict the efficiencies of maintenance methylation, de novo methylation and hydroxylation over time. In our modeling we analyzed both aggregated and single CpG behavior for each locus. Both average and single CpG modeling gave similar results. The single CpG data, summarized in the supplementary information (see S3 and S4 Figs), gave slightly increased confidence intervals compared to averaged data. In our further analysis we use averaged data for model interpretation.

**Fig 5:**
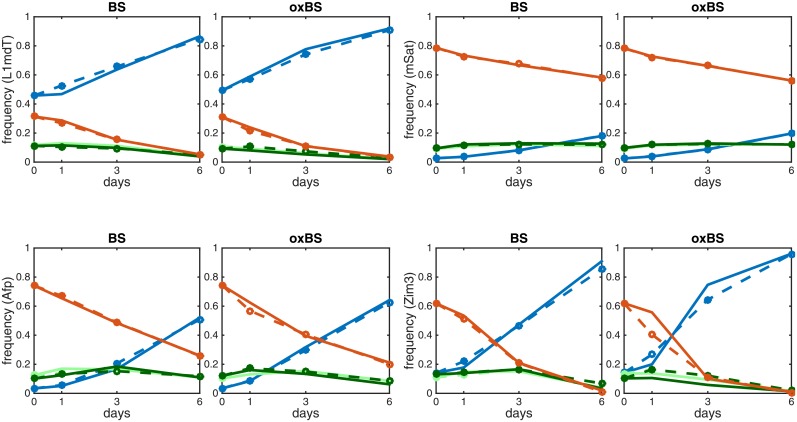
Comparison of predicted modification levels and the obtained sequencing data for BS and oxBS for the loci L1mdT (top-left), mSat (top-right), Afp (bottom-left), Zim3 (bottom-right); TT (blue), TC (light green), CT (dark green), CC (red). The solid lines show the experimentally measured frequencies states and the dashed lines correspond to the values predicted by the two HMMs.

Using the estimated values of the model’s unknown parameters we could predict the probabilities of the observable states and compare them to the measured data at various time points. The model accurately describes the dynamics for all loci except for some underestimations of two states CC and TT for oxBs in Ttc25 and Zim3, respectively. (Fig 5 and S1 Fig).

**Fig 6:**
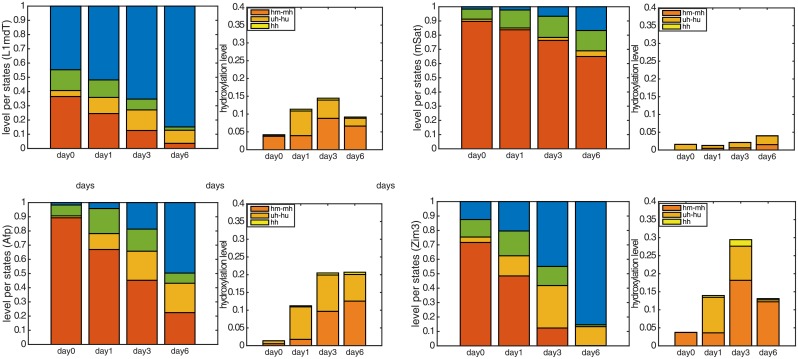
Probabilities of the hidden states for L1mdT (top-left), mSat (top-right), Afp (bottom-left) and Zim3 (bottom-right): The left diagram depicts the amount of fully methylated (*mm*) sites in red color, hemimethylated (*um* and *mu*) sites in green color, and unmethylated (*uu*) sites in blue color. The orange block gives the total amount of CpG sites with at least one 5hmC(hidden states), while the detailed distribution of the hydroxylated states is given by the diagram on the right.

Fig 6 shows the probabilities of the hidden states in L1mdT, mSat, Afp, and Zim3, where the parameters are chosen according to the results of the maximum likelihood estimation. The left bar diagram shows the probabilities of all fully methylated (*mm*), hemimethylated (*um* and *mu*) and unmethylated (*uu*) sites, as well as the total amount of the hydroxylated CpG dyads, i.e., those containing at least one 5hmC. The detailed level of all hydroxylated sites is depicted in the right diagram.

From previous experiments it was known that 5hmC levels initially increase during cultivation in 2i [21, 22]. However, precise levels had not been determined per locus. Our analysis provides the first accurate locus specific determination of 5hmC changes. Our estimation of 5hmC confirms an initial increase of hydroxylated cytosines over time for most loci besides L1mdA and Snrpn. L1mdA shows a low level of 5mC and 5hmC, which only slightly decreases in 2i. Snrpn also shows a relatively low level of 5mC and a non significant amount of 5hmC, which do not change in 2i over time (S2 Fig). The highest hydroxylation levels are found in the single copy genes Zim3 and Afp with a maximum level of 0.30 and 0.20. For Afp, mSat, IAP and MuERVL (see Fig 6 and S2 Fig), the maximum hydroxylation level is seen at day6, while for L1mdT, Ttc25 and Zim3 at day3. The latter can be explained by the particularly low 5mC levels between day3 and day6 in these loci which naturally reduces the potential substrates for the Tet enzymes. However, the level of 5hmC (orange bar in Fig 6 and S2 Fig, left) relative to the total modification level (5hmC + 5mC) (red, orange and green bars), becomes maximal on the sixth day for all loci that show a loss of 5mC. This points towards an increasingly important role of 5hmC in the loss of methylation over time.

Indeed, the probability *p* (see HMM subsection) that a 5hmC site is not recognized by Dnmt1 (or the Dnmt1/Uhrf1 complex), which corresponds to states (*hu*) and (*uh*) in the model, is estimated to be 1 with very small standard deviations for all the loci that show significant 5hmC levels. We estimated smaller values for *p* only for those loci where hydroxylation is nearly absent (mSat, MuERVL, Snrpn).

**Fig 7:**
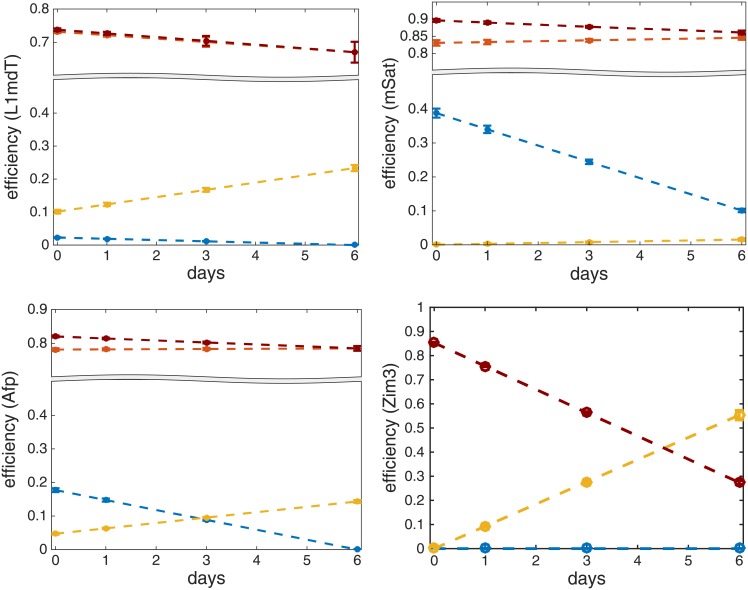
The diagrams show the enzymatic efficiencies and their standard deviations for maintenance (red), de novo (blue), hydroxylation (yellow) and total efficiency on a hemimethylated CpG (dark red). Results are given for L1mdT (top-left), mSat (top-right), Afp (bottom-left) and Zim3 (bottom-right) over time.

In Fig 7 we plot the functions *μ*_*m*_(*t*), *μ*_*d*_(*t*), *η*(*t*) and λ(*t*) over time together with their estimated standard deviations. Note that the estimated standard deviations of all the efficiencies are very small (maximum half width of all confidence intervals is 0.031). For the exact estimates and their standard deviations see S3 and S4 Tables. From the above efficiencies we can deduce the impact of de novo methylation activity on the hemimethylated dyads as the difference between the total methylation efficiency and maintenance methylation, i.e., $\lambda \left(t\right)-\mu_{m}\left(t\right)={\bar{\mu }}_{m}\left(t\right)\cdot \mu_{d}\left(t\right)$ (see Fig 7). Our data indicates that persistence of DNA methylation at Afp, mSat, IAP and MuERVL elements clearly depends also on de novo enzymes acting on hemimethylated CpGs.

For each efficiency, we performed a statistical test with a confidence level of 1% for the null hypothesis that the slope of the corresponding linear function is zero, i.e., that the efficiencies are constant over time (see in addition S1 Text). Furthermore, to eliminate the possibility of overfitting due to the linear assumption, we performed leave-one-out cross-validation (LOOCV) to estimate the test error of our model with constant efficiencies against a linear model. Results in S5 Table show that the linear assumption improves the prediction up to 38.3%. Further tests concerning the sensitivity of the model w.r.t. the parameters showed that the model is also sufficiently robust (see S1 Text).

Overall, the estimation of the efficiency functions reveals some common and some locus specific features that accompany the DNA demethylation dynamics over time in 2i. As a common feature we observe that the total methylation on hemimethylated sites, λ(*t*), decreases over time in all examined loci but at different rates. Along with this decrease we observe also a drop of de novo methylation activity at all loci besides Ttc25 and Zim3. In contrast, hydroxylation activity increases for most loci over time (except for Snrpn). Interestingly, the largest increase of *η*(*t*) occurs in L1mdT and the two DMRs in the genes Ttc25 and Zim3, where we also observe low or even total absence of de novo activity. On the other hand, a weaker hydroxylation activity in mSat, as well as IAP and MuERVL (S2 Fig), is accompanied by a strong decrease of *μ*_*d*_(*t*) in the same loci, while in Afp both de novo methylation and hydroxylation show a moderate decrease and increase, respectively. At last, maintenance methylation seems to differ among loci. For all repetitive multicopy loci and Afp maintenance activity remains nearly constant while for Ttc25 and Zim3 it shows a significant decrease. For the imprinted Snrpn locus, where the methylation level remains constant, our model accurately predicts the apparently constantly high maintenance efficiency of 1.0. Altogether, these findings point towards a major impairment of maintenance methylation by 5hmC. Additionally, for each locus this impairment is modulated by a distinct combination of decreasing (e.g. Dnmt3a,b) or increasing (e.g. Tet) activities in a locus specific manner. Some of the locus specific differences may also have their origin in the particular methylation and (hydroxy-)methylation status present in serum/LIF before the shift into 2i.

## Discussion

The goal of our study was to investigate the role of 5hmC in the process of progressive DNA demethylation at single copy and mulitcopy loci across the genome. As a model system we used the DNA of ES cells grown under conditions where the cells experience a genome wide reduction of DNA methylation [21, 22].

Using time dependent comparative bisulfite and oxidative bisulfite hairpin sequencing data we generated two HMMs: one that represents the dynamics of total modifications (5mC and 5hmC in BS) and the other only representing the 5mC levels (in oxBS). The comparison allowed us to accurately determine the amount and changes of 5hmC at certain genomic loci, to estimate the transient distribution of both 5mC and 5hmC in the DNA and to compute statistically reliable estimates for the efficiencies of maintenance and de novo methylation, as well as for hydroxylation over time.

Our first finding is that 5hmC changes over time and can be modeled along with the overall changes in symmetric DNA methylation at CpGs. Our estimates give us an exact knowledge of 5hmC dynamics, which is congruent with the finding that several Tet enzymes are up-regulated in 2i medium [21, 22]. The calculation of the hidden state probabilities and the efficiencies of the different enzyme-driven processes show that the 5hmC dependent demethylation rates differ considerably from locus to locus. However, the dynamics of the (hydroxy-)methylation levels for the CpGs of the same locus show a certain homogeneity (see S3 and S4 Figs).

The second major finding is that loci with an enrichment of 5hmC such as Afp, L1mdT and IAP show higher demethylation rates compared to mSat or Snrpn. Hence, 5hmC containing DNA strands are indeed more likely to lose DNA methylation over time. Our modeling strongly supports the hypothesis that 5hmC is less well recognized by the maintenance methylation machinery (Dnmt1/Uhrf1 complex) as indicated by the estimation of the corresponding non-recognition probability *p*. The accumulation of 5hmC then causes a passive dilution mechanism of CpG methylation with each DNA replication/cell cycle, despite of the fact that the model predicts a constant behavior of maintenance activity in most of the analyzed loci. In ES cells maintained in 2i medium this mechanism appears to be the main driving force for a rapid and linear DNA demethylation.

Interestingly, in contrast to the previously shown unchanged mRNA expression of Dnmt1 and Uhrf1 in 2i [21, 22] we observe a strong decrease of maintenance function for the single copy genes Ttc25 and Zim3 (see Fig 7 and S2 Fig, red line). Since the influence of 5hmC on the maintenance mechanism is reflected by the recognition probability *p*, the observed decrease is independent of the high 5hmC levels at these loci. This indicates an additional impairment or absence of the maintenance machinery at these loci. However, we cannot exclude the possibility that with the strong decrease in maintenance efficiency our model, at least to some extent, compensates for active demethylation which we cannot capture with our current experimental/model design.

Being able to estimate the de novo methylation impact of Dnmt3a/b on hemimethylated sites, the third observation of our model is that all analyzed elements show a compromised de novo methylation activity as an additional factor contributing to an enhanced local DNA demethylation. The predicted behavior for the involved enzymes’ activities appears to follow their relative expression in 2i medium, in which both Dnmt3a and Dnmt3b are clearly down regulated [21, 22]. Our observations, thus, suggest that the down regulation of Dnmt3a and Dnmt3b activities appears to enhance the 5hmC dependent CpG demethylation. This may be either directly due to a decreased methylation efficiency on hemimethylated sites or due to a lower abundance of the enzymes.

In summary, we present a novel HMM method that allows to precisely measure and describe effects related to the influence of 5hmC on the persistence of DNA methylation in the mammalian genome. The modeling allows us to decipher complex DNA methylation patterns and enables us to accurately infer enzymatic activities. In its current form the model already captures a fraction of possible demethylation dynamics and scenarios most likely reflecting many loci in the genome. A genome wide application of our modeling is possible. It comes, though, at the expense of locus specific accuracy since with the existing whole genome hairpin sequencing methods data is difficult to generate and will not reach a sufficient sequencing depth. However, our approach can also be used to accurately model 5hmC dependent methylation dynamics in diseases, e.g. certain cancers and in aging processes of long lived cells. By integrating novel precise sequencing methods, which detect other oxidized modifications the model can be enhanced to additionally capture active demethylation and describe the involved processes.

## Supporting Information

S1 Text Details on the theoretical model, the parameter estimation, the statistical analysis of the results and the experimental procedure. (PDF)

S1 Fig Comparison of model prediction and data for IAP, L1mdA, MuERVL, Ttc25 and Snrpn. Plotted according to Fig 5.
(PDF)

S2 Fig Results for loci IAP, L1mdA, MuERVL, Ttc25 and Snrpn. Left: Probabilities of the hidden states. Plotted according to Fig 6. Right: Estimated efficiencies and standard deviations over time. Plotted according to Fig 7
(PDF)

S3 Fig Estimated efficiencies and standard deviations for each single CpG dyad of repetitive elements IAP, L1mdA, L1mdT, mSat, MuERVL and single copy genes Afp, Ttc25, Zim3 and Snrpn over time. In the case of IAP we cover six CpG positions. However, during evolution CpG one and five underwent a transition resulting in a loss of the CpG positions in this particular IAP class. Furthermore, due to the lack of space we only show the first 6 CpGs out of 13, (8) CpGs, analyzed in L1mdA, (Zim3). The colormap is the same as in Fig 7.
(PDF)

S4 Fig (Hydroxy-)methylation levels for each single CpG dyad of repetitive elements IAP, L1mdA, L1mdT, mSat, MuERVL and single copy genes Afp, Ttc25, Zim3 and Snrpn over time. The colormap is the same as in Fig 6.
(PDF)

S1 Table BS and oxBS data, Conversion Errors (repetitive elements). (PDF)

S2 Table BS and oxBS data, Conversion Errors (single copy genes). (PDF)

S3 Table Estimated coefficients of the functions *μ*_*d*_(*t*), *μ*_*m*_(*t*) and *η*(*t*) and their approximate standard deviations. The p-values have been taken conducting a hypothesis test *H*_0_: *β*_1_ = 0 using the Wald statistic.
(PDF)

S4 TableEstimated coefficients of the function λ(*t*) and their approximate standard deviations.The p-values have been taken conducting a hypothesis test $H_{0}:\beta_{1}^{\lambda }=0\wedge \beta_{2}^{\lambda }=0$ using the Wald statistic.(PDF)

S5 Table Test error: Linear vs Constant Assumption. Computed Kullback-Leibler divergence and Bhattacharya distance values given by LOOCV data to compare the test error for assuming linear vs constant efficiencies.
(PDF)

S1 Dataset Bs and oxBS data for single CpGs. The dataset contains the BS and oxBS data of each single CpG and loci after processing of the sequencing data; error rates are included as separate files. Raw sequencing data are available on request.
(ZIP)
