# Supplementary material for: The Influence of Hydroxylation on Maintaining CpG Methylation Patterns: A Hidden Markov Model Approach
Source: PLoS Comput Biol. 2016 May 25;12(5):e1004905. doi: 10.1371/journal.pcbi.1004905 (PMC4880293; doi:10.1371/journal.pcbi.1004905)
Supplement: S1 Text — (PDF) [file pcbi.1004905.s001.pdf]

S1 Text: The Influence of Hydroxylation on Maintaining CpG Methylation Patterns: a Hidden Markov Model Approach (with tables A-D)

P. Giehr, C. Kyriakopoulos, G. Ficz, V. Wolf, J. Walter

BS and oxBS Data

In Tables S1, S2 we show the data for the DNA loci L1mdA, L1mdT, IAP, mSat, MuERVL, Afp, Ttc25, Zim3 and Snrpn taken from bisulfite and oxidative bisulfite sequencing together with the measured conversion errors $\bar{c}$, $\bar{d}$, $\bar{e}$ and $\bar{f}$ for each locus. The conversion errors are calculated using the hairpin linker which is ligated onto the DNA[1]. A more detailed description of the conversion errors' calculation is given in Section 4.1. The measurement times are: 24h after incubation on Serum (day0), and 24h (day1), 72h (day3) and 144h (day6) on 2i. Each table shows the total number of CpGs of the corresponding locus that have been observed in each of the four observable states (TT, TC, CT and CC) for every day of measurerement.

Estimation of model parameters

Initial distribution of the hidden states

Let $\pi(0)$ be the unknown initial distribution of the hidden states and let $\pi(i,t) = P(\mathcal{X}(t) = i)$ represent the entry of $\pi(t)$ that corresponds to state $i \in S$. In addition, denote by $n_{bs}(j,t)$ and $n_{ox}(j,t)$ the number of times that state $j \in \mathcal{S}_{obs}$ has been observed during independent BS and oxidative BS measurements at time t.

We want to solve the problem: $\pi(0)^* = \arg\max_{\pi(0)} \mathcal{L}_1(\pi(0))$, subject to the constraint $\sum_{i \in \mathcal{S}} \pi(i,0) = 1$, where

$$\mathcal{L}_1(\pi(0)) = \prod_{j \in \mathcal{S}_{obs}} \pi_{bs}(j,0)^{n_{bs}(j,0)} \cdot \pi_{ox}(j,0)^{n_{ox}(j,0)}.$$

We consider the log-likelihood

$$\log \mathcal{L}_1(\pi(0)) = \sum_{j \in \mathcal{S}_{obs}} n_{bs}(j,0) \cdot \log \pi_{bs}(j,0) + n_{ox}(j,0) \cdot \log \pi_{ox}(j,0)).$$

For a gradient descent optimization procedure we need its derivative w.r.t. $\pi(0)$ given by

$$\frac{d}{d\pi(0)} \log \mathcal{L}_1(\pi(0)) = \sum_{j \in \mathcal{S}_{obs}} n_{bs}(j,0) \cdot \frac{\frac{d}{d\pi(0)} \pi_{bs}(j,0)}{\pi_{bs}(j,0)} + n_{ox}(j,0) \cdot \frac{\frac{d}{d\pi(0)} \pi_{ox}(j,0)}{\pi_{ox}(j,0)}.$$

[1] For Snprn we had to use a hairpin linker without 5mC or 5hmC and therefore could not calculate the sample specific conversion error. Instead we applied the mean errors of all other loci analyzed in this study.

Let $\pi_{bs}(t), \pi_{ox}(t)$ be the vectors with entries $\pi_{bs}(j,t), \pi_{ox}(j,t),\ \ \forall j \in \mathcal{S}_{obs}, \forall t \in T_{obs}$. Writing the derivatives $\frac{d}{d\pi(0)}\pi_{bs}(j,0)$ and $\frac{d}{d\pi(0)}\pi_{ox}(j,0)$ in vector-matrix notation we get

$$\frac{d}{d\pi(0)}\pi_{bs}(0) = \frac{d}{d\pi(0)}\pi(0) \cdot \mathbf{E}_{bs}(0) = \mathbf{E}_{bs}(0), \quad \frac{d}{d\pi(0)}\pi_{ox}(0) = \frac{d}{d\pi(0)}\pi(0) \cdot \mathbf{E}_{ox}(0) = \mathbf{E}_{ox}(0),$$

which gives us the gradient of the log-likelihood function w.r.t. the initial distribution of the hidden states after insertion into the above equation.

Estimation of the efficiencies

Let $\mathbf{v} = (\beta_0^{\mu_m}, \beta_1^{\mu_m}, \beta_0^{\mu_d}, \beta_1^{\mu_d}, \beta_0^{\eta}, \beta_1^{\eta}, p)$, be the vector of the seven unknown parameters where μ_m stands for maintenance, μ_d for de novo and η for hydroxylation efficiency, while p is the probability that 5hmC is not considered during maintenance. Recall that we assume that the efficiencies are linear functions of time and so $\mathbf{v}$ contains the coefficients of these functions. E.g. $\mu_m(t) = \beta_0^{\mu_m} + t \cdot \beta_1^{\mu_m}$. The transition matrix of the discrete Markov chain at time unit t is $\mathbf{P}(t) = \mathbf{D}(t) \cdot \mathbf{M}(t) \cdot \mathbf{H}(t)$, where

$$\mathbf{D}(t) = \begin{array}{c} \\ uu \\ um \\ mu \\ uh \\ hu \\ hm \\ mh \\ mm \\ hh \end{array} \begin{array}{c} \begin{array}{ccccccccc} uu & um & mu & uh & hu & hm & mh & mm & hh \end{array} \\ \left(\begin{array}{ccccccccc}
1 & 0 & 0 & 0 & 0 & 0 & 0 & 0 & 0 \\
1/2 & 1/2 & 0 & 0 & 0 & 0 & 0 & 0 & 0 \\
1/2 & 0 & 1/2 & 0 & 0 & 0 & 0 & 0 & 0 \\
1/2 & 0 & 0 & 1/2 & 0 & 0 & 0 & 0 & 0 \\
1/2 & 0 & 0 & 0 & 1/2 & 0 & 0 & 0 & 0 \\
0 & 1/2 & 0 & 0 & 1/2 & 0 & 0 & 0 & 0 \\
0 & 0 & 1/2 & 1/2 & 0 & 0 & 0 & 0 & 0 \\
0 & 1/2 & 1/2 & 0 & 0 & 0 & 0 & 0 & 0 \\
0 & 0 & 0 & 1/2 & 1/2 & 0 & 0 & 0 & 0
\end{array}\right) \end{array},$$

$$\mathbf{M}(t) \begin{array}{c} \\ uu \\ um \\ mu \\ uh \\ hu \\ hm \\ mh \\ mm \\ hh \end{array} \begin{array}{c} \begin{array}{ccccccccc} uu & um & mu & uh & hu & hm & mh & mm & hh \end{array} \\ \left(\begin{array}{ccccccccc}
\bar{\mu}_d^2 & \mu_d \cdot \bar{\mu}_d & \mu_d \cdot \bar{\mu}_d & 0 & 0 & 0 & 0 & \mu_d^2 & 0 \\
0 & \bar{\lambda} & 0 & 0 & 0 & 0 & 0 & \lambda & 0 \\
0 & 0 & \bar{\lambda} & 0 & 0 & 0 & 0 & \lambda & 0 \\
0 & 0 & 0 & p \cdot \bar{\mu}_d + \bar{p} \cdot \bar{\lambda} & 0 & 0 & p \cdot \mu_d + \bar{p} \cdot \lambda & 0 & 0 \\
0 & 0 & 0 & 0 & p \cdot \bar{\mu}_d + \bar{p} \cdot \bar{\lambda} & p \cdot \mu_d + \bar{p} \cdot \lambda & 0 & 0 & 0 \\
0 & 0 & 0 & 0 & 0 & 1 & 0 & 0 & 0 \\
0 & 0 & 0 & 0 & 0 & 0 & 1 & 0 & 0 \\
0 & 0 & 0 & 0 & 0 & 0 & 0 & 1 & 0 \\
0 & 0 & 0 & 0 & 0 & 0 & 0 & 0 & 1
\end{array}\right) \end{array}$$

and

$$\mathbf{H}(t) = \begin{array}{c} \\ uu \\ um \\ mu \\ uh \\ hu \\ hm \\ mh \\ mm \\ hh \end{array} \begin{array}{c} \begin{array}{ccccccccc} uu & um & mu & uh & hu & hm & mh & mm & hh \end{array} \\ \left(\begin{array}{ccccccccc}
1 & 0 & 0 & 0 & 0 & 0 & 0 & 0 & 0 \\
0 & \bar{\eta} & 0 & \eta & 0 & 0 & 0 & 0 & 0 \\
0 & 0 & \bar{\eta} & 0 & \eta & 0 & 0 & 0 & 0 \\
0 & 0 & 0 & 1 & 0 & 0 & 0 & 0 & 0 \\
0 & 0 & 0 & 0 & 1 & 0 & 0 & 0 & 0 \\
0 & 0 & 0 & 0 & 0 & \bar{\eta} & 0 & 0 & \eta \\
0 & 0 & 0 & 0 & 0 & 0 & \bar{\eta} & 0 & \eta \\
0 & 0 & 0 & 0 & 0 & \eta \cdot \bar{\eta} & \eta \cdot \bar{\eta} & \bar{\eta}^2 & \eta^2 \\
0 & 0 & 0 & 0 & 0 & 0 & 0 & 0 & 1
\end{array}\right) \end{array}.$$

Note that for $\mathbf{D}(t)$ we can omit the time parameter t since it is time-independent.

Given, now, $\pi(0)$, we want to compute the maximum likelihood estimator (MLE) $\mathbf{v}^* = \mathrm{argmax}_{\mathbf{v}} \log\mathcal{L}_2(\mathbf{v})$, where

$$\mathcal{L}_2(\mathbf{v}) = \prod_{t \in T_{obs} \setminus \{0\}} \prod_{j \in \mathcal{S}_{obs}} \pi_{bs}(j,t)^{n_{bs}(j,t)} \cdot \pi_{ox}(j,t)^{n_{ox}(j,t)}.$$

	bisulfite sequencing				ox. bisulfite sequencing			
	TT	TC	CT	CC	TT	TC	CT	CC
uu	c^2	$c\cdot\bar{c}$	$c\cdot\bar{c}$	$\bar{c}^2$	c^2	$c\cdot\bar{c}$	$c\cdot\bar{c}$	$\bar{c}^2$
um	$c\cdot\bar{d}$	$c\cdot d$	$\bar{c}\cdot\bar{d}$	$\bar{c}\cdot\bar{d}$	c^2	$c\cdot\bar{c}$	$c\cdot\bar{c}$	$\bar{c}^2$
mu	$c\cdot d$	$\bar{c}\cdot\bar{d}$	$c\cdot d$	$\bar{c}\cdot d$	$c\cdot\bar{d}$	$\bar{c}\cdot\bar{d}$	$c\cdot d$	$\bar{c}\cdot d$
uh	$c\cdot\bar{e}$	$c\cdot e$	$\bar{c}\cdot\bar{e}$	$\bar{c}\cdot e$	$c\cdot f$	$c\cdot\bar{f}$	$\bar{c}\cdot f$	$\bar{c}\cdot\bar{f}$
hu	$c\cdot\bar{e}$	$\bar{c}\cdot\bar{e}$	$c\cdot e$	$\bar{c}\cdot e$	$c\cdot f$	$\bar{c}\cdot f$	$c\cdot\bar{f}$	$\bar{c}\cdot\bar{f}$
hm	$\bar{d}\cdot\bar{e}$	$d\cdot\bar{e}$	$\bar{d}\cdot e$	$d\cdot e$	$\bar{d}\cdot f$	$d\cdot f$	$\bar{d}\cdot\bar{f}$	$\bar{d}\cdot f$
mh	$\bar{d}\cdot\bar{e}$	$\bar{d}\cdot e$	$d\cdot\bar{e}$	$d\cdot e$	$\bar{d}\cdot f$	$\bar{d}\cdot\bar{f}$	$d\cdot f$	$\bar{d}\cdot f$
mm	$\bar{d}^2$	$\bar{d}\cdot d$	$d\cdot\bar{d}$	d^2	$\bar{d}^2$	$\bar{d}\cdot d$	$d\cdot\bar{d}$	d^2
hh	$\bar{e}^2$	$\bar{e}\cdot e$	$e\cdot\bar{e}$	e^2	f^2	$f\cdot\bar{f}$	$f\cdot\bar{f}$	$\bar{f}^2$

Table A: Transition probabilities from hidden to the observable states in BS and in oxBS.

The only constraint for the above problem is that the efficiencies should be probabilities for all the considered time points, i.e., $0 \leq \beta_0 + \beta_1 \cdot t \leq 1,\ \forall t \in \{0, 6\}$ for all the efficiencies, and the same constraint holds for p, i.e., $0 \leq p \leq 1$.

It holds

$$\log \mathcal{L}_2(\mathbf{v}) = \sum_{t\in T_{obs}\setminus\{0\}} \sum_{j\in\mathcal{S}_{obs}} n_{bs}(j,t)\cdot \log \pi_{bs}(j,t) + n_{ox}(j,t)\cdot \log \pi_{ox}(j,t)$$

and we get the score vector of the log-likelihood function as

$$\frac{d}{d\mathbf{v}} \log \mathcal{L}_2(\mathbf{v}) = \sum_{t\in T_{obs}\setminus\{0\}} \sum_{j\in\mathcal{S}_{obs}} n_{bs}(j,t)\cdot \frac{\frac{d}{d\mathbf{v}}\pi_{bs}(j,t)}{\pi_{bs}(j,t)} + n_{ox}(j,t)\cdot \frac{\frac{d}{d\mathbf{v}}\pi_{ox}(j,t)}{\pi_{ox}(j,t)}.$$

Then the matrix-vector form of the derivatives $\frac{d}{d\mathbf{v}}\pi_{bs}(j,t)$ and $\frac{d}{d\mathbf{v}}\pi_{ox}(j,t)$ can be written as

$$\frac{d}{d\mathbf{v}}\pi_{bs}(t) = \frac{d}{d\mathbf{v}}\pi(t)\cdot \mathbf{E}_{bs}(t) \ \text{ and } \ \frac{\mathrm{d}}{\mathrm{d}\mathbf{v}}\pi_{\mathrm{ox}}(\mathrm{t}) = \frac{\mathrm{d}}{\mathrm{d}\mathbf{v}}\pi(\mathrm{t})\cdot \mathbf{E}_{\mathrm{ox}}(\mathrm{t}),\ \ \forall \mathrm{t} \in \mathrm{T}_{\mathrm{obs}},$$

where the entries of the emission matrices $\mathbf{E}_{bs}(t)$ and $\mathbf{E}_{ox}(t)$ are given in Table A.

Considering, now, the forward Kolmogorov equation for the discrete Markov chain and its derivative w.r.t. the parameters it suffices to simultaneously solve the following two equation systems.

$$\begin{aligned} \pi(t) &= \pi(t-1)\cdot \mathbf{P}(t) \\ \frac{d}{d\mathbf{v}}\pi(t) &= \frac{d}{d\mathbf{v}}\pi(t-1)\cdot \mathbf{P}(t) + \pi(t-1)\frac{d}{d\mathbf{v}}\mathbf{P}(t),\ \ \forall t \geq 1 \end{aligned} \quad (1)$$

with $\frac{d}{d\mathbf{v}}\pi(0) = 0$ and $\pi(0) = \pi(0)^*$. The derivative of the transition matrix is

$$\frac{d}{d\mathbf{v}}\mathbf{P}(t) = \frac{d}{d\mathbf{v}}(\mathbf{D}\cdot\mathbf{M}(t)\cdot\mathbf{H}(t)) = \mathbf{D}\cdot\left(\frac{d}{d\mathbf{v}}\mathbf{M}(t)\cdot\mathbf{H}(t) + \mathbf{M}(t)\cdot\frac{d}{d\mathbf{v}}\mathbf{H}(t)\right)$$

E.g. applying the chain rule and writing μ_m instead of $\mu_m(\beta_0^{\mu_m}, \beta_1^{\mu_m}, t)$ we get

$$\frac{d}{d\beta_0^{\mu_m}}\mathbf{M}(\mu_m) = \frac{d}{d\mu_m}\mathbf{M}(\mu_m)\cdot\frac{d}{d\beta_0^{\mu_m}}\mu_m = \frac{d}{d\mu_m}\mathbf{M}(\mu_m)$$

and

$$\frac{d}{d\beta_1^{\mu_m}}\mathbf{M}(\mu_m) = \frac{d}{d\mu_m}\mathbf{M}(\mu_m)\cdot\frac{d}{d\beta_1^{\mu_m}}\mu_m = \frac{d}{d\mu_m}\mathbf{M}(\mu_m)\cdot t.$$

In the same way we get the first derivatives w.r.t. all the other components of parameter vector $\mathbf{v}$. Applying once more the product rule in Eq. (1), and using similar arguments as above we can also compute the second partial derivatives $\frac{d}{d\mathbf{v}_i d\mathbf{v}_j} \log \mathcal{L}_2(\mathbf{v})$ which will give us the (i,j)-th entry of the Hessian matrix $\mathcal{H} = \nabla\nabla^{\mathrm{T}} \log \mathcal{L}_2(\mathbf{v})$.

Standard deviations and confidence intervals

The observed Fisher information is defined as $\mathcal{J}(\mathbf{v}^*) = -\mathcal{H}(\mathbf{v}^*)$, where $\mathbf{v}^*$ is the maximum likelihood estimator. The expected Fisher information is $\mathcal{I}(\mathbf{v}) = \mathbb{E}[\mathcal{J}(\mathbf{v})]$ and its inverse is a lower bound for the covariance matrix of the MLE. Thus, here we approximate the standard deviations of the estimates as $\sigma(\mathbf{v}^*) = \sqrt{\mathrm{Var}(\mathbf{v}^*)} = \sqrt{\mathrm{diag}(-\mathcal{H}^{-1}(\mathbf{v}^*))}$. In order to approximate the standard deviations of the efficiencies over time, i.e. $\sigma(\mu_m(t)), \sigma(\mu_d(t))$ and $\sigma(\eta(t))$, we exploit the fact that if $f(t) = \beta_0 + \beta_1 \cdot t$ then $\mathrm{Var(f(t))} = \mathrm{Var}(\beta_0 + \beta_1 \cdot \mathrm{t}) = \mathrm{Var}(\beta_0) + \mathrm{t}^2\mathrm{Var}(\beta_1) + 2\mathrm{tCov}(\beta_0, \beta_1)$.

Given, now, the variances of the estimated efficiencies we can compute the variance $\lambda(t)$, for any t as

$$\mathrm{Var}(\lambda) = \mathrm{Var}(\mu_\mathrm{m}) + \mathrm{Var}(\mu_\mathrm{d}) + 2\mathrm{Cov}(\mu_\mathrm{m}, \mu_\mathrm{d}) + \mathrm{Var}(\mu_\mathrm{m}\mu_\mathrm{d}) - 2\mathrm{Cov}(\mu_\mathrm{m}, \mu_\mathrm{m}\mu_\mathrm{d}) - 2\mathrm{Cov}(\mu_\mathrm{m}, \mu_\mathrm{m}\mu_\mathrm{d}),$$

where the last four terms are computed as follows:

$$\mathrm{Cov}(\mu_\mathrm{m}, \mu_\mathrm{d}) = \mathrm{Cov}(\beta_0^{\mu_\mathrm{m}}, \beta_0^{\mu_\mathrm{d}}) + \mathrm{tCov}(\beta_0^{\mu_\mathrm{m}}, \beta_1^{\mu_\mathrm{d}}) + \mathrm{tCov}(\beta_1^{\mu_\mathrm{m}}, \beta_0^{\mu_\mathrm{d}}) + \mathrm{t}^2\mathrm{Cov}(\beta_1^{\mu_\mathrm{m}}, \beta_1^{\mu_\mathrm{d}}),$$

and

$$\mathrm{Var}(\mu_\mathrm{m}\mu_\mathrm{d}) = \mathbb{E}[\mu_\mathrm{m}^2\mu_\mathrm{d}^2] - \mathbb{E}[\mu_\mathrm{m}\mu_\mathrm{d}]^2 \quad (2)$$

$$\mathrm{Cov}(\mu_\mathrm{m}, \mu_\mathrm{m}\mu_\mathrm{d}) = \mathbb{E}[\mu_\mathrm{m}^2\mu_\mathrm{d}] - \mathbb{E}[\mu_\mathrm{m}]\mathbb{E}[\mu_\mathrm{m}\mu_\mathrm{d}], \quad (3)$$

$$\mathrm{Cov}(\mu_\mathrm{d}, \mu_\mathrm{m}\mu_\mathrm{d}) = \mathbb{E}[\mu_\mathrm{d}^2\mu_\mathrm{m}] - \mathbb{E}[\mu_\mathrm{d}]\mathbb{E}[\mu_\mathrm{m}\mu_\mathrm{d}] \quad (4)$$

Since the MLEs are approximately normally distributed and for any two random variables X, Y, $\mathbb{E}[XY] = \mathrm{Cov(X,Y)} + \mathbb{E}[\mathrm{X}]\mathbb{E}[\mathrm{Y}]$, we get

$$\mathbb{E}[\mu_m^2\mu_d] = E[\mu_m]^2E[\mu_d] + \mathrm{Var}(\mu_\mathrm{m})\mathrm{E}[\mu_\mathrm{d}] + 2\mathrm{Cov}(\mu_\mathrm{m}, \mu_\mathrm{d})\mathrm{E}[\mu_\mathrm{m}]$$

$$\mathbb{E}[\mu_d^2\mu_m] = E[\mu_d]^2E[\mu_m] + \mathrm{Var}(\mu_\mathrm{d})\mathrm{E}[\mu_\mathrm{m}] + 2\mathrm{Cov}(\mu_\mathrm{m}, \mu_\mathrm{d})\mathrm{E}[\mu_\mathrm{d}]$$

$$\begin{aligned}\mathbb{E}[\mu_m^2\mu_d^2] \quad = \quad & E[\mu_m]^2E[\mu_d]^2 + \mathrm{Var}(\mu_\mathrm{m})\mathrm{Var}(\mu_\mathrm{d}) + \mathrm{Var}(\mu_\mathrm{d})\mu_\mathrm{m}^2 + \mathrm{Var}(\mu_\mathrm{m})\mathrm{E}[\mu_\mathrm{d}]^2 \\ & +2\mathrm{Cov}(\mu_\mathrm{m}, \mu_\mathrm{d})^2 + 4\mathrm{Cov}(\mu_\mathrm{m}, \mu_\mathrm{d})\mathrm{E}[\mu_\mathrm{m}]\mathrm{E}[\mu_\mathrm{d}],\end{aligned}$$

where the expectations and thus all terms in Eq. (2) - (4) are now known. Obtaining this way the standard deviations of all the efficiencies over time one can create the corresponding confidence intervals for a fixed confidence level, here $\beta = 95\%$ was chosen. For instance the confidence interval for the total methylation on hemimethylated sites will be

$$\lambda \pm z \cdot \sigma(\lambda) = \lambda \pm z \cdot \sqrt{\mathrm{Var}(\lambda)},$$

where $z = F^{-1}\left(\frac{\beta+1}{2}\right)$ and F is the cummulative distribution function (cdf) of the standard normal distribution. Similarly we get the confidence intervals for all remaining parameters.

Hypothesis Test

We carried out a number of hypothesis tests related to the estimated parameters (for the results see Section 3). Here we briefly describe the details of the Wald test which is the one we used here to validate our results.

Given a maximum likelihood estimate $\mathbf{v}^*$ of an unknown parameter vector $\mathbf{v}_0 \in V \subseteq \mathbb{R}^p$ we want to test the null hypothesis H_0 that $g(\mathbf{v}_0) = 0$, where $g : \mathbb{R}^p \to \mathbb{R}^r$ is a vector valued function with $r \leq p$. We define the Wald statistic for this estimate as

$$w = g(\mathbf{v}^*)^\intercal \left[J_g(\mathbf{v}^*) \cdot \widehat{\Sigma} \cdot J_g(\mathbf{v}^*)^\intercal\right]^{-1} g(\mathbf{v}^*),$$

where $J_g(\mathbf{v}^*)$ is the Jacobian of g, i.e., the $r \times p$ matrix of the partial derivatives of the entries of g with respect to the entries of $\mathbf{v}$, and $\widehat{\Sigma}$ is a consistent estimate of the assymptotic covariance matrix, here equal to the negative Hessian, of $\mathbf{v}^*$. Note that w here is a realization of a random variable $W_{\mathbf{v}^*}$ as it is a function of $\mathbf{v}^*$ which is a random variable itself depending on the observed data.

Under the regularity assumptions that for all $\mathbf{v} \in V$, the entries of g are continuously differentiable w.r.t. all entries of $\mathbf{v}$ and that $J_g(\mathbf{v})$ has rank r, the following holds. If the null hypothesis is true, i.e. $g(\mathbf{v}_0) = 0$, then the Wald statistic $W_{\mathbf{v}^*}$ converges to a Chi-square distribution with r degrees of freedom [4].

Thus, conducting the Wald test consists of comparing the Wald statistic with a critical threshold $z = F^{-1}(1 - a)$, where F is the cdf of a Chi-square random variable with r degrees of freedom and a is a predefined significance level, e.g. $a = 1\%$. If $w > z$ then we can safely reject the null hypothesis. The p-value of the test is the probability p $= P(W_{\mathbf{v}^*} > w) = 1 - P(W_{\mathbf{v}^*} \leq w) \approx 1 - F(w)$ and so equivalently one also rejects the null hypothesis if p $\leq a$.

For estimates taken from maximum likelihood alternative tests, such as likelihood ratio or score test, are also possible. The Wald statistic, however, is convenient in case of testing multiple hypotheses in parallel. In addition, the use of all tests mentioned before for our estimates returned similar p-values and did not lead to a different result regarding the cases that one rejects H_0.

Results

In S3 Table we present the MLEs returned by our global optimization routine for the parameter vector $\mathbf{v}$ and the corresponding vector of standard deviations $\sigma(\mathbf{v})$, given the data of Section 1 for each of the nine genome loci. The p-value of the efficiencies μ_m, μ_d and η corresponds to the null hypothesis $H_0 : \beta_1 = 0$, where β_1 is the gradient of the corresponding efficiency, and for the total methylation λ it takes the form $H_0 : \beta_1^\lambda = 0 \wedge \beta_2^\lambda = 0$, since λ is a quadratic function of time. The significance level α for deciding H_0 for each of the above parameters has been set to 1%. S4 Table shows the computed coefficients of the total methylation $\lambda(t)$, which can be implicitly taken from the maintenance and de novo estimated coefficients.

In S1 Figure we see the predicted probabilities of the observable states that have been taken using the estimated values of S3 Table for each region. We compare them to the measured data (transformed to frequencies) of S1 and S2 Tables at the various days. S2 Figure shows the predicted probabilities of the hidden states and the detailed hydroxylation levels, as well as the estimated (hydroxy-)methylation efficiencies over time for the loci IAP, L1mdA, MuERVL, Ttc25 and Snrpn that do not appear in the manuscript.

From the performed Wald test we found a statistically significant decrease for the de novo, and the total methylation efficiencies in all nine loci (besides de novo at Ttc25, Zim3 and Snrpn where it is absent and total methylation in L1mdA). Similarly, the increase of hydroxylation for five out of nine loci is statistically significant. However, for the maintenance function we have to accept the null hypothesis in most of the loci (namely all repetitive elements and Afp), that is, we cannot exclude the possibility that for these loci maintenance is constant over time.

In order to measure the test error of the model we performed leave-one-out cross-validation (LOOCV) and tested two competing assumptions: 1)" The enzyme efficiencies are constant" and 2)" The enzyme efficiencies can also be linear". For each locus we tested the prediction of the model for each single CpG, having trained it on the data of the other CpGs and we averaged at the end the test error. For comparing the prediction ability of the model for each of the two cases 1) and 2) we

used two different distribution distance measures (Kullback-Leibler divergence and Bhattacharyya distance) between the data distribution P and the predicted by the model distribution Q. Kullback-Leibler (KL) divergence is defined as $D_{KL}(P||Q) = \sum_i P(i) \log \frac{P(i)}{Q(i)}$ and the Bhattacharyya distance as $BC(P,Q) = -\log\left(\sum_i \sqrt{P(i)Q(i)}\right)$, where i goes here over the observable states.

Our results in S5 Table show that for all loci the test error, i.e., the above distance, becomes evidently smaller for the case where we allow efficiencies to be linear over time. In the two columns where we report the improvement ("gain") $\frac{\text{KL-const - KL-linear}}{\text{KL-const}}$ of the test error, we see that the decrease of the test error using the linear model over the constant varies from 0.6% (in mSat) to 38.3% (in Zim3) for the Kullback-Leibler distance. The predictive potential of the model, and consequently the above gain ratio, depends on the available number of CpGs for the training data and on how much the efficiencies deviate from constant behavior over time.

In S3 Fig., S4 Fig. we show the (hydroxy-)methylation efficiencies and the (hydroxy-)methylation levels for all CpGs of all the examined loci, in case the data of each locus is not aggregated and separate estimations are taken for each of the single CpG dyads. Allthough the absolute (hydroxy-)methylation levels at distinct CpGs can be slightly different, one observes that the tendency of the demethylation process has clearly homogeneous characteristics between CpGs of the same locus. Particularly, the increase of the hydroxylation level in relation to the methylated substrates is always present. Also, the day with the highest absolute 5hmC level is, in the majority of the cases, the same for the CpGs of a locus. Similarly, the predicted behavior of the enzymes' efficiencies within a locus is in principle homogeneous with some differences in the absolute estimated values that come with larger confidence intervals due to the smaller number of samples.

Finally, to validate the robustness of the model sensitivity analysis of the parameters has been examined. Perturbing one parameter at a time (OAT) by $\pm 1\%$ we get a maximum (over all loci, time points and parameters) absolute change of 0.0053 for the total hydroxylation level and 0.0198 for the total methylation level.

Hairpin oxidative Bisulfite Sequencing

500 ng of mESC DNA was cleaved with 10 units of restriction enzymes for 5h in a 30 µl reaction. For IAP L1mdA the DNA was cut with DdeI (New England Biolabs; NEB), for mSat and MuERVL with Eco47I (Thermo Fisher Scientific), Afp, Ttc25, Zim3 with TaqI (Thermo Fisher Scientific) and in case of Snrpn with NlaIII (NEB). The restriction was stopped by a 20 min heat inactivation at 80°C. The restricted DNA was then subjected to a 16 h or overnight ligation with T4-DNA Ligase (New England Biolabs). 200 units of T4-DNA Ligase, 4 µl 10mM ATP and 1µl 100 µM hairpin linker was added directly into the restriction reaction and the volume was adjusted to 40 µl using ddH2O. During ligation the hairpin linker becomes covalent attached to the restriction site of the DNA. Purification and oxidative BS treatment was carried out using the chemicals and protocols provided by Cambridge Epigenetix. Amplicons were generated by PCR using Hotfire Taq polymerase from Solis Biodyne. Sequencing was carried out using the MiSeq Illumina system (paired end sequencing 2x250bp reads). After Seqecning in a first informatics step the adapter sequence is removed from the reads (Trimming). The resulting read information is then analyzed analyzed using the BiQAnalyzerHT and a python script. For the repeats the sequences were filtered by sequence identity score, meaning that only reads which matched the reference sequence to at least 80% were used for the analysis. For single copy genes this score was set to 90% and in addition only reads with maximum 10% missing CpG sites were analyzed.

Primer- and Reference sequences

Table B shows the sequence of the nine different hairpin linkers used to covalent link both DNA strands. We included unmodified cytosine, 5mC(X) and 5hmC(y) into the hairpin linker to follow the conversion of these modifications during BS and oxBS treatment. Mapping the sequencing information to this reference

Table B: Sequence of the hairpin linker for Afp, L1mdT, L1mdA, mSat, IAP; *M* indicates the localization of 5mC, *H* the position of 5hmC in the sequence. All hairpin linker carry a 5'-phosphorylation.

Hairpin	Linker Sequence
IAP-HP	*Pho*-TNAGGG*M*CCATDDDDDDDDDATGGG*H*CC
L1mdA-HP	*Pho*-TNAGGG*M*CCATDDDDDDDDDATGGG*H*CC
L1mdT-HP	*Pho*-CCGGAGGG*M*CCATDDDDDDDDDATGGG*H*CCT
mSat-HP	*Pho*-GNCGGG*M*CCATDDDDDDDDDATGGG*H*CC
MuERVL-HP	*Pho*-GNCGGG*M*CCATDDDDDDDDDATGGG*H*CC
Afp-HP	*Pho*-CGGGG*M*CCATDDDDDDDDDATGGG*H*CC
Ttc25-HP	*Pho*-CGGGG*M*CCATDDDDDDDDDATGGG*H*CC
Zim3-HP	*Pho*-CGGGG*M*CCATDDDDDDDDDATGGG*H*CC
Snrpn-HP	*Pho*-GGGCCTADDDDDDDDDTAGGCCCCATG

Table C: Primer for amplification of the analyzed loci after bisulfite and oxidative bisulfite treatment.

Primer	Sequence
IAP-HP-Forward	TTTTTTTTTTAGGAGAGTTATATTT
IAP-HP-Revers	ATCACTCCCTAATTAACTACAAC
L1mdA-HP-Forward	GTGAGTGGATTATAGTGTTTGTTTTAA
L1mdA-HP-Revers	AAATAAATCACAATACCTACCCCAAT
L1mdT-HP-Forward	TGGTAGTTTTTAGGTGGTATAGAT
L1mdT-HP-Revers	TCAAACACTATATTACTTTAACAATTCCCA
mSat-HP-Forward	GGAAAATTTAGAAATGTTTAATGTAG
mSat-HP-Revers	AACAAAAAAACTAAAAATCATAAAAA
MuERVL-HP-Forward	TAAGGGTTAGGTGGTAGTATTGAAT
MuERVL-HP-Revers	CAAAAACCAAATAACAACATTAAAT
Afp-HP-Forward	TTTTGTTATAGGAAAATAGTTTTTAAGTTA
Afp-HP-Revers	AAATCACAAAACATCTTACCTATCC
Ttc25-HP-Forward	TGAAAGAGAATTGATAGTTTTTAGG
Ttc25-HP-Revers	AAAACAAAAATCTATTCCATCACTC
Zim3-HP-Forward	TTTATTTATTTGTGTGTGGTTTTTG
Zim3-HP-Revers	CACATATCAAAATCCACTCACCTAT
Snrpn-HP-Forward	AGAATTTATAAGTTTAGTTGATTTTTT
Snrpn-HP-Revers	TAATCAAATAAAATACACTTTCACTACT

sequences we determine the states of each cytosine which allows us to calculate all possible measurement errors for each time point and each genomic region. For example: 5hmC should be converted after oxBS treatment to 5fU and will after sequencing seen as T. We check for each sequenced hairpin molecule the state of the 5hmC position which can be either C or T. We divide then the number of T by the total number of T and C at this position to get the conversion error of 5hmC during oxBS treatment. The conversion error for cytosine and 5mC is calculated in the same way. For Snrpn we had to use a hairpin linker without 5mC or 5hmC and could therefore not calculate the conversion errors for this sample probably. However, to correct for more general errors we used the mean conversion error of all other loci. In addition table C and table D give the primer sequences and the corresponding reference sequence for each regions respectively.

IAP	TGTCACTCCCTGATTGGCTGCAGCCCATCGGCCGAGTTGACGTCACGGGGAAGGCAGAGCACATGGAGTAGAGAACCACCCTC<br>GGCATATGCGCAGATTATTTGTTTACCAC**TNAGGGMCCATDDDDDDDDATGGGHCC**TAAGTGGTAAACAAATAATCTGCGCAT<br>ATGCCGAGGGTGGTTCTCTACTCCATGTGCTCTGCCTTCCCCGTGACGTCAACTCGGCCGATGGGCTGCAGCCAATCAGGGAG<br>TGACA
L1mdA	TCCAATCGCGCGGAACTTGAGACTGCGGTACATAGGGAAGCAGGCTACCCGGGCCTGATCTGGGGCACAAGTCCCTTCCGCTC<br>GACTCGAGACTCGAGCCCCGGGCTACCTTGCCAGCAGAGTCTTGCCCAACACCCGCAAGGGCCCACACGGGACTCCCCACGGG<br>ACCC**TNAGGGMCCATDDDDDDDDATGGGHCC**TNAGGGTCCCGTGGGGAGTCCCGTGTGGGCCCTTGCGGGTGTTGGGCAAGAC<br>TCTGCTGGCAAGGTAGCCCGGGGCTCGAGTCTCGAGTCGAGCGGAAGGGACTTGTGCCCCAGATCAGGCCCGGGTAGCCTGCT<br>TCCCTATGTACCGCAGTCTCAAGTTCCGCGCGATTGGATTGGGGCAGGCACTGTGATCCACTC
L1mdT	CCCGGGACCAAGATGGCGACCGCTGCTGCTGTGGCTTAGGCCGCCTCCCCAGCCGGGTGGGCACCTGT<br>CCT**CCGGAGGGMCCATDDDDDDDDATGGGHCCT**CCGGAGGACAGGTGCCCACCCGGCTGGGGAGGCGG<br>CCTAAGCCACAGCAGCAGCGGTCGCCATCTTGGTCCCGGG
mSat	GGAAAATTTAGAAATGTTTAATGTAGGACGTGGAATATGGCAAGAAAACTGAAAATCATGGGAAATGA<br>GAAACATCCACTTGTCGACTTGAAAAATGACGAAATCACTAAAAAACGTGAAAAATGAGAAATGCACA<br>CTGAAG**GWCGGGMCCATDDDDDDDDATGGGHCC**GWCCTTCAGTGTGCATTTCTCATTTTTCACGTTTT<br>TTAGTGATTTCGTCATTTTTCAAGTCGACAAGTGGATGTTTCTCATTTTTTATGATTTTTAGTTTTTT<br>TGTT
MuERVL	CGCCCGAGACAAGGTGATTCTAGTTATTATAATGGACAGCGTAGACAAAAGAATGTTTATAATAACAT<br>ACCCAGTAATGGTCAGCACAGGAGAGGTGAAATTTATAATGGCATGACTCGGTTG**GWCGGGMCCATDD**<br>**DDDDDDATGGGHCC**GWTTCAACCGAGTCATGCCATTATAAATTTCACCTCTCCTGTGCTGACCATTAC<br>TGGGTATGTTATTATAAACATTCTTTTGTCTACGCTGTCCATTATAATAACTAGAATCACCTTGTCTC<br>GGGCG
Afp	TTTTGTTATAGGAAAATAGTTTTTAAGTTACAAAGCATCTTACCTATCCCAAACTCATTTTCGTGCAA<br>TGCTTTGGACGCAGCGAAATGTAGCAGGAGGATGAGGGAAGCGGGTGTGATCCACTTCATGGCTGCTG<br>GTTCCTTCACCGCAGGCAGTGCTGGAAGTGGGATGTTT**CGGGGMCCATDDDDDDDDATGGGHCC**CGAA<br>ACATCCCACTTCCAGCACTGCCTGCGGTGAAGGAACCAGCAGCCATGAAGTGGATCACACCCGCTTCC<br>CTCATCCTCCTGCTACATTTCGCTGCGTCCAAAGCATTGCACGAAAATGAGTTTGGGATAGGTAAGAT<br>GtTTTGTGATTT
Ttc25	CCAGTAGATCCTCAGCTGGGGGCAGGGATCTATTCCATCACTCCCCTTCCGTGTCGGGATTTCGTGCA<br>GCTCAGACGGGTCCAAGTCTTACACAAGCTGTCCTAACTGCTGTGCGTTTATATAACAACTACCCGGT<br>TGTGTTTAGAAAACACTGTTTT**CGGGGMCCATDDDDDDDDATGGGHCC**CGAAAACAGTGTTTTCTAAA<br>CACAACCGGGTAGTTGTTATATAAACGCACAGCAGTTAGGACAGCTTGTGTAAGACTTGGACCCGTCT<br>GAGCTGCACGAAATCCCGACACGGAAGGGGAGTGATGGAATAGATCCCTGCCCC
Zim3	CCCGGCCACCATAGTCGGATTATCCGTGGGCGGGGTGAGATGGACGGAGCGCCTTGCAGACCTCAGGA<br>AAACCTCCCCACGCCTGTCCGGCCTTGGCTTGGTGACAGGGAAACTGGCTGGACT**CGGGGMCCATDDD**<br>**DDDDDATGGGHCC**CGAGTCCAGCCAGTTTCCCTGTCACCAAGCCAAGGCCGGACAGGCGTGGGGAGGT<br>TTTCCTGAGGTCTGCAAGGCGCTCCGTCCATCTCACCCCGCCCACGGATAATCCGACTATGGTGGCCG<br>GGCAAGGACCACAC
Snrpn	AGAATTTACAAGTTTAGTTGATTTTTTTCGCTCCATTGCGTTGCAAATCACTCCTCAGAACCAAGCGT<br>CTGGCATCTCCGGCTCCCTCTCCTCTCTGCGCTAGTCTTGCCGCAATGGCTCAGGTTTGTCGCGCGGC<br>TCCCTACGCATG**GGGCCTADDDDDDDDTAGGCCCCATG**CGTAGGGAGCCGCGCGACAAACCTGAGCCA<br>TTGCGGCAAGACTAGCGCAGAGAGGAGAGGGAGCCGGAGATGCCAGACGCTTGGTTCTGAGGAGTGAT<br>TTGCAACGCAATGGAGCGAGGAAGGTCAGCTGGGCTTGTGGATTCTAGTAGTGAAAGTGTATTTTATT<br>TGATTA

Table D: Reference Sequences used for 5mC and 5hmC analysis; M = 5mC, H = 5hmC

References

[1] A. Andreychenko, L. Mikeev, D. Spieler, and V. Wolf. Approximate maximum likelihood estimation for stochastic chemical kinetics. *EURASIP Journal on Bioinformatics and Systems Biology*, 2012(1):1–14, 2012.

[2] J. K. Knight. *Rotation points from motion capture data using a closed form solution*. PhD thesis, University of Colorado at Colorado Springs, 2008.

[3] Y. Pawitan. *In all likelihood: statistical modelling and inference using likelihood*. Oxford University Press, 2001.

[4] M. Taboga. *Lectures on probability theory and mathematical statistics*. CreateSpace Independent Pub., 2012.
