# Supplementary figures and images for: The Influence of Hydroxylation on Maintaining CpG Methylation Patterns: A Hidden Markov Model Approach
Source: PLoS Comput Biol. 2016 May 25;12(5):e1004905. doi: 10.1371/journal.pcbi.1004905 (PMC4880293; doi:10.1371/journal.pcbi.1004905)

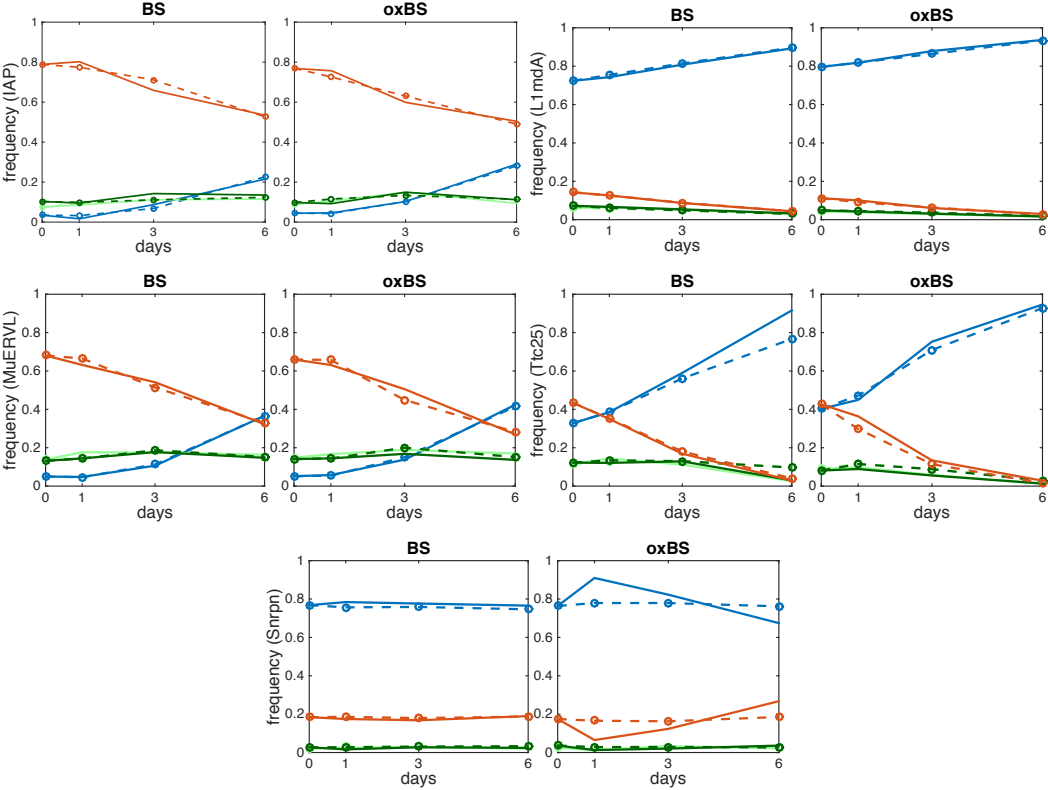

Supplement: S1 Fig — Plotted according to Fig 5. (PDF) [file pcbi.1004905.s002.pdf]

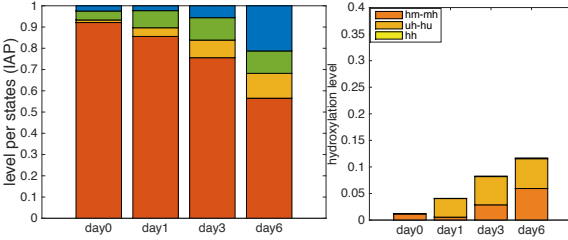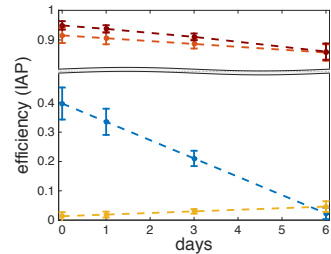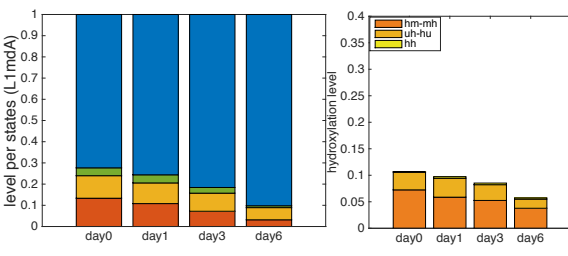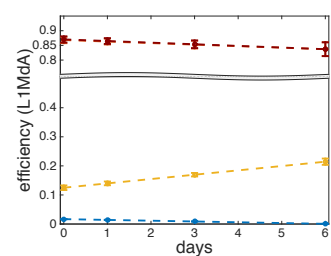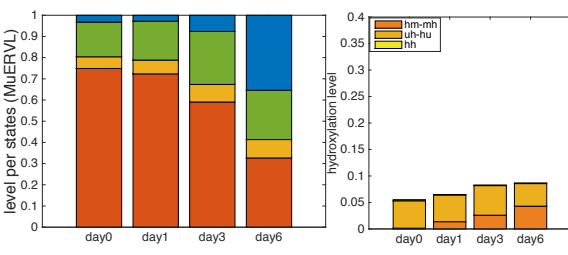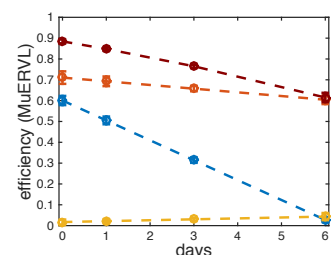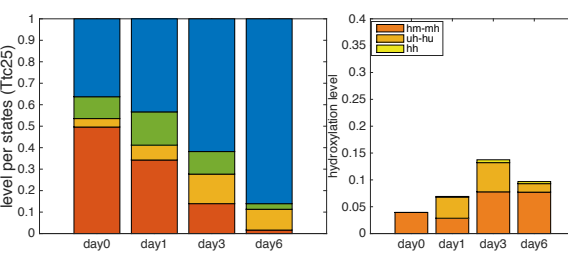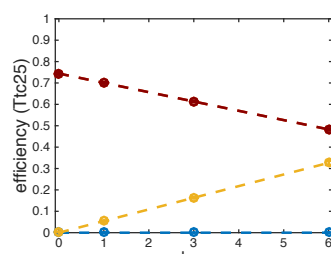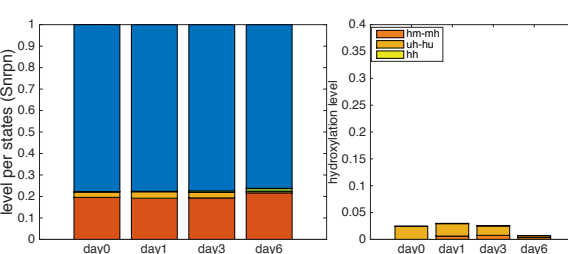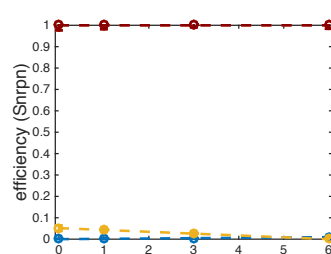

Supplement: S2 Fig — Left: Probabilities of the hidden states. Plotted according to Fig 6. Right: Estimated efficiencies and standard deviations over time. Plotted according to Fig 7 (PDF) [file pcbi.1004905.s003.pdf]
