# Supplementary material for: The Influence of Hydroxylation on Maintaining CpG Methylation Patterns: A Hidden Markov Model Approach
Source: PLoS Comput Biol. 2016 May 25;12(5):e1004905. doi: 10.1371/journal.pcbi.1004905 (PMC4880293; doi:10.1371/journal.pcbi.1004905)
Supplement: S3 Fig — In the case of IAP we cover six CpG positions. However, during evolution CpG one and five underwent a transition resulting in a loss of the CpG positions in this particular IAP class. Furthermore, due to the lack of space we only show the first 6 CpGs out of 13, (8) CpGs, analyzed in L1mdA, (Zim3). The colormap is the same as in Fig 7. (PDF) [file pcbi.1004905.s004.pdf]

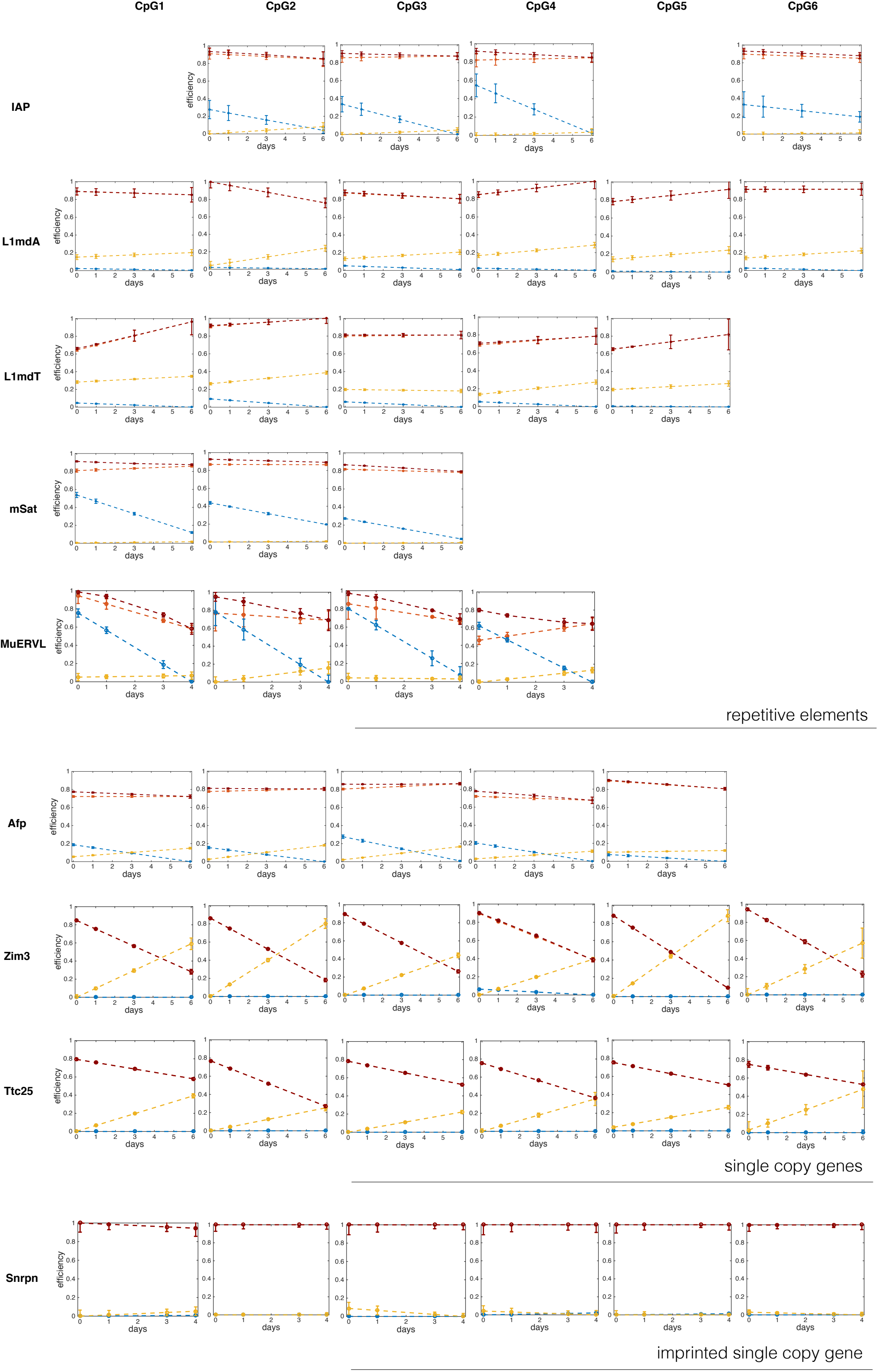
CpG1
CpG2
CpG3
CpG4
CpG5
CpG6
IAP
L1mdA
L1mdT
mSat
MuERVL
repetitive elements
Afp
Zim3
Ttc25
single copy genes
Snrpn
imprinted single copy gene
efficiency
days
