# Supplementary material for: The Influence of Hydroxylation on Maintaining CpG Methylation Patterns: A Hidden Markov Model Approach
Source: PLoS Comput Biol. 2016 May 25;12(5):e1004905. doi: 10.1371/journal.pcbi.1004905 (PMC4880293; doi:10.1371/journal.pcbi.1004905)
Supplement: S4 Fig — The colormap is the same as in Fig 6. (PDF) [file pcbi.1004905.s005.pdf]

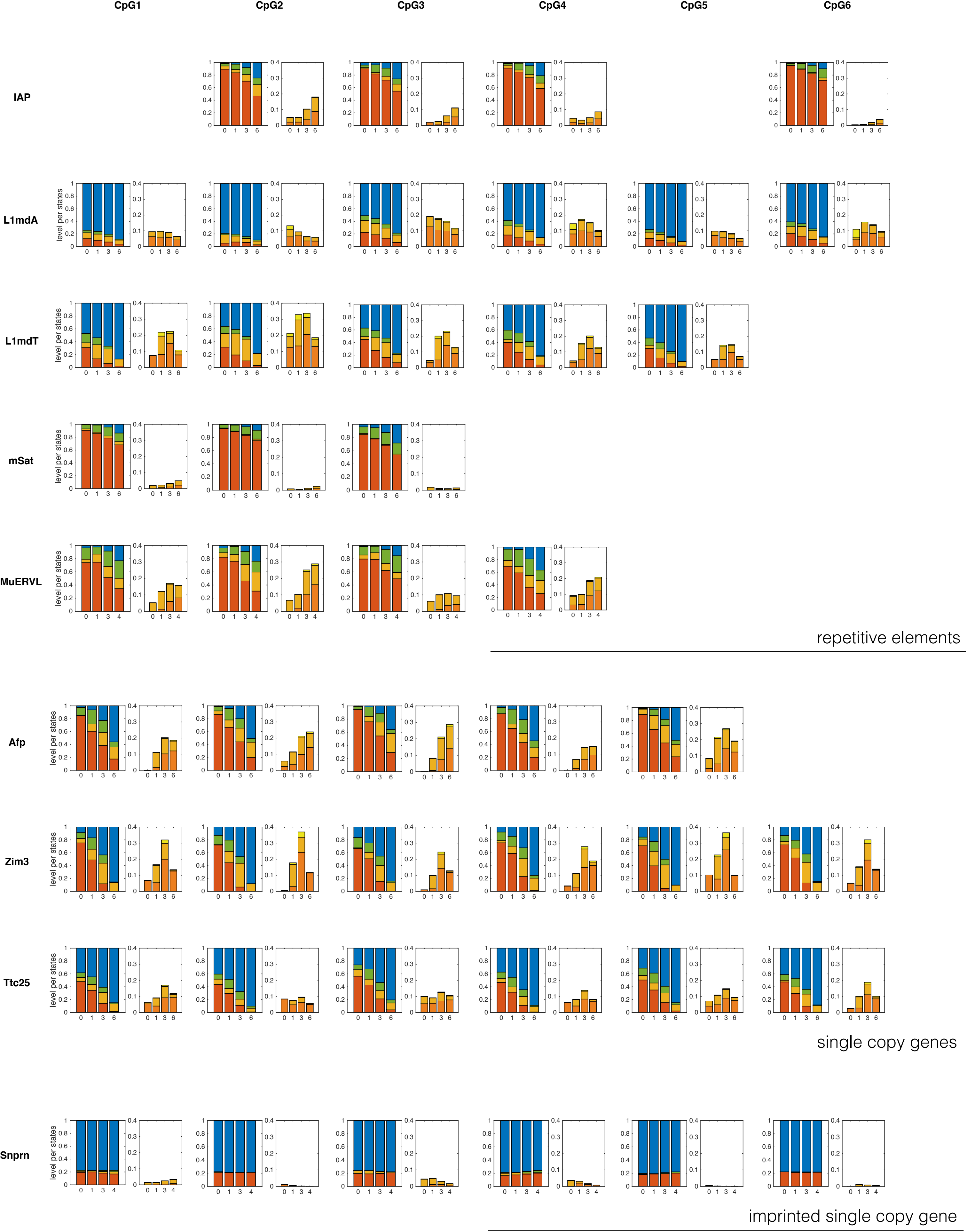
CpG1
CpG2
CpG3
CpG4
CpG5
CpG6
IAP
L1mdA
L1mdT
mSat
MuERVL
level per states
repetitive elements
Afp
Zim3
Ttc25
single copy genes
Snprn
imprinted single copy gene
