# Supplementary material for: The Influence of Hydroxylation on Maintaining CpG Methylation Patterns: A Hidden Markov Model Approach
Source: PLoS Comput Biol. 2016 May 25;12(5):e1004905. doi: 10.1371/journal.pcbi.1004905 (PMC4880293; doi:10.1371/journal.pcbi.1004905)
Supplement: S1 Table — (PDF) [file pcbi.1004905.s006.pdf]

IAP

	BS							oxBS						
day	TT	TC	CT	CC	$\bar{c}$	$\bar{d}$	$\bar{e}$	TT	TC	CT	CC	$\bar{c}$	$\bar{d}$	$\bar{f}$
0	39	84	116	890	0.003	0.0709	0.0774	35	70	77	605	0.002	0.0935	0.0701
1	17	89	99	831	0.002	0.0685	0.0411	57	131	115	943	0.002	0.0813	0.0939
3	68	87	111	513	0.001	0.0628	0.0721	77	112	112	449	0.001	0.09	0.0905
6	283	152	178	703	0.003	0.0829	0.0455	210	68	81	365	0.002	0.0737	0.0942

L1mdA

	BS							oxBS						
day	TT	TC	CT	CC	$\bar{c}$	$\bar{d}$	$\bar{e}$	TT	TC	CT	CC	$\bar{c}$	$\bar{d}$	$\bar{f}$
0	41088	3479	4106	8092	0.006	0.0795	0.0734	36286	1968	2203	5094	0.004	0.0853	0.0016
1	30095	2607	2697	5118	0.006	0.078	0.0645	32774	1555	1715	4026	0.004	0.0845	0.0015
3	44382	2819	2953	4769	0.005	0.084	0.0736	35886	1175	1293	2486	0.004	0.0795	0.0913
6	75920	2627	2762	3731	0.005	0.0841	0.0685	54132	965	979	1699	0.004	0.0897	0.083

L1mdT

	BS							oxBS						
day	TT	TC	CT	CC	$\bar{c}$	$\bar{d}$	$\bar{e}$	TT	TC	CT	CC	$\bar{c}$	$\bar{d}$	$\bar{f}$
0	37715	9668	9192	25857	0.007	0.0802	0.0739	30511	6368	5713	19208	0.005	0.0784	0.0729
1	41882	11690	10300	25648	0.008	0.0887	0.0743	43459	6807	5923	17638	0.004	0.0780	0.0738
3	44766	7868	6875	10804	0.007	0.0880	0.0703	31379	2470	2125	4419	0.006	0.0758	0.0683
6	44687	2154	2023	2758	0.006	0.0807	0.0758	56830	1363	1263	2352	0.005	0.0856	0.0714

mSat

	BS							oxBS						
day	TT	TC	CT	CC	$\bar{c}$	$\bar{d}$	$\bar{e}$	TT	TC	CT	CC	$\bar{c}$	$\bar{d}$	$\bar{f}$
0	492	1676	1738	14403	0.004	0.0718	0.0567	315	1170	1221	9804	0.004	0.0663	0.0772
1	448	1337	1495	9029	0.005	0.073	0.0666	568	1678	1748	10654	0.004	0.0727	0.0698
3	1288	1926	2043	10540	0.004	0.0685	0.0642	1171	1602	1697	8746	0.003	0.0685	0.0631
6	3625	2248	2570	11757	0.004	0.0738	0.0605	2618	1619	1604	7471	0.003	0.0725	0.0722

MuERVL

	BS							oxBS						
day	TT	TC	CT	CC	$\bar{c}$	$\bar{d}$	$\bar{e}$	TT	TC	CT	CC	$\bar{c}$	$\bar{d}$	$\bar{f}$
0	111	307	293	1516	0.007	0.0801	0.0695	108	309	295	1381	0.005	0.0646	0.0800
1	149	553	452	1978	0.007	0.0745	0.0632	238	689	597	2607	0.003	0.0492	0.0649
3	448	735	746	2276	0.007	0.1123	0.0740	345	471	420	1262	0.003	0.1321	0.0687
6	798	356	321	702	0.009	0.0606	0.0582	1458	584	470	927	0.003	0.0772	0.0951
