# Supplementary material for: The Influence of Hydroxylation on Maintaining CpG Methylation Patterns: A Hidden Markov Model Approach
Source: PLoS Comput Biol. 2016 May 25;12(5):e1004905. doi: 10.1371/journal.pcbi.1004905 (PMC4880293; doi:10.1371/journal.pcbi.1004905)
Supplement: S2 Table — (PDF) [file pcbi.1004905.s007.pdf]

Afp

day	BS TT	TC	CT	CC	$\bar{c}$	$\bar{d}$	$\bar{e}$	oxBS TT	TC	CT	CC	$\bar{c}$	$\bar{d}$	$\bar{f}$
0	1401	5233	4235	31088	0.004	0.0854	0.0852	1208	3652	4307	26568	0.005	0.0982	0.0728
1	2022	6718	4946	25945	0.007	0.0636	0.0646	2821	4367	5366	20886	0.004	0.0836	0.0616
3	4917	4884	5453	14311	0.004	0.0674	0.0765	11285	5443	4739	14034	0.004	0.0636	0.0800
6	29537	6220	6222	14733	0.005	0.0888	0.0523	22516	2989	2182	7421	0.004	0.0638	0.0593

Ttc25

day	BS TT	TC	CT	CC	$\bar{c}$	$\bar{d}$	$\bar{e}$	oxBS TT	TC	CT	CC	$\bar{c}$	$\bar{d}$	$\bar{f}$
0	16873	5945	6297	22363	0.07	0.0726	0.0751	19490	4338	3926	20641	0.005	0.077	0.1023
1	17013	6342	5340	15431	0.07	0.0625	0.0341	20389	4448	4042	16499	0.006	0.0725	0.0577
3	26107	4950	5705	7472	0.06	0.0813	0.0785	34016	2630	2501	6059	0.004	0.1078	0.058
6	19121	538	627	595	0.06	0.0762	0.059	44122	570	619	1310	0.005	0.0686	0.0933

Zim3

day	BS TT	TC	CT	CC	$\bar{c}$	$\bar{d}$	$\bar{e}$	oxBS TT	TC	CT	CC	$\bar{c}$	$\bar{d}$	$\bar{f}$
0	14479	11308	13448	63716	0.005	0.065	0.0755	1777	1695	1285	7754	0.007	0.1388	0.1047
1	14295	11947	11222	43046	0.003	0.0717	0.0575	11829	8157	6249	33002	0.007	0.0958	0.0835
3	31291	10020	10965	13864	0.005	0.0666	0.0647	38515	4875	2983	5202	0.008	0.0807	0.0663
6	112883	4761	4100	2434	0.005	0.076	0.0707	1132054	503	457	345	0.006	0.0616	0.0871

Snrpn

day	BS TT	TC	CT	CC	$\bar{c}$	$\bar{d}$	$\bar{e}$	oxBS TT	TC	CT	CC	$\bar{c}$	$\bar{d}$	$\bar{f}$
0	3092	83	109	742	0.0133	0.0757	0.0733	2620	86	125	599	0.0044	0.0785	0.0690
1	3183	100	67	709	0.0135	0.0725	0.0582	3497	48	49	250	0.0038	0.0742	0.0601
3	2571	92	91	557	0.0116	0.0789	0.0717	3357	136	84	503	0.0038	0.0855	0.0731
6	3098	82	98	768	0.0121	0.0779	0.06131	2377	77	127	943	0.0039	0.0759	0.0799
