# Supplementary material for: The Influence of Hydroxylation on Maintaining CpG Methylation Patterns: A Hidden Markov Model Approach
Source: PLoS Comput Biol. 2016 May 25;12(5):e1004905. doi: 10.1371/journal.pcbi.1004905 (PMC4880293; doi:10.1371/journal.pcbi.1004905)
Supplement: S3 Table — The p-values have been taken conducting a hypothesis test H0: β1 = 0 using the Wald statistic. (PDF) [file pcbi.1004905.s008.pdf]

IAP: (hydroxy) methylation prob.	β_0	$\sigma(\beta_0)$	β_1	$\sigma(\beta_1)$	p-value
μ_m	0.9155	0.0256	-0.0097	0.0072	0.180
μ_d	0.3977	0.0545	-0.0624	0.0106	$< 10^{-5}$
η	0.0134	0.0132	0.0055	0.0045	0.226
p	1	0.2577	-	-	-

L1mdA: (hydroxy) methylation prob.	β_0	$\sigma(\beta_0)$	β_1	$\sigma(\beta_1)$	p-value
μ_m	0.8682	0.0104	-0.0052	0.0040	0.190
μ_d	0.0168	0.0007	-0.0027	0.0002	$< 10^{-5}$
η	0.1249	0.0074	0.0149	0.0023	$< 10^{-5}$
p	1	0.0238	-	-	-

L1mdT: (hydroxy) methylation prob.	β_0	$\sigma(\beta_0)$	β_1	$\sigma(\beta_1)$	p-value
μ_m	0.7317	0.0040	-0.0102	0.0044	0.020
μ_d	0.0229	0.0010	-0.0038	0.0002	$< 10^{-5}$
η	0.1013	0.0046	0.0220	0.0015	$< 10^{-5}$
p	1	0.0468	-	-	-

mSat: (hydroxy) methylation prob.	β_0	$\sigma(\beta_0)$	β_1	$\sigma(\beta_1)$	p-value
μ_m	0.8304	0.0080	0.0026	0.0019	0.186
μ_d	0.3879	0.0133	-0.0478	0.0025	$< 10^{-5}$
η	0.0002	0.0038	0.0026	0.0011	0.024
p	0.8025	0.1966	-	-	-

MuERVL: (hydroxy) methylation prob.	β_0	$\sigma(\beta_0)$	β_1	$\sigma(\beta_1)$	p-value
μ_m	0.7106	0.0300	-0.0177	0.0076	0.019
μ_d	0.6006	0.0221	-0.0955	0.0039	$< 10^{-5}$
η	0.0172	0.0119	0.0044	0.0045	0.336
p	0.5428	0.2858	-	-	-

Afp: (hydroxy) methylation prob.	β_0	$\sigma(\beta_0)$	β_1	$\sigma(\beta_1)$	p-value
μ_m	0.7817	0.0041	0.0006	0.0015	0.717
μ_d	0.1772	0.0058	-0.0295	0.0011	$< 10^{-5}$
η	0.0473	0.0028	0.0160	0.0010	$< 10^{-5}$
p	1	0.0208	-	-	-

Ttc25: (hydroxy) methylation prob.	β_0	$\sigma(\beta_0)$	β_1	$\sigma(\beta_1)$	p-value
μ_m	0.7440	0.0064	-0.0435	0.0003	$< 10^{-5}$
μ_d	0.0000	0.0018	-0.0000	0.0003	1
η	0.0000	0.0072	0.0544	0.0023	$< 10^{-5}$
p	1	0.0670	-	-	-

Zim3: (hydroxy) methylation prob.	β_0	$\sigma(\beta_0)$	β_1	$\sigma(\beta_1)$	p-value
μ_m	0.8530	0.0027	-0.0965	0.0014	$< 10^{-5}$
μ_d	0.0000	0.0022	-0.0000	0.0005	1
η	0.0000	0.0087	0.0922	0.0047	$< 10^{-5}$
p	1	0.0255	-	-	-

Snrpn: (hydroxy) methylation prob.	β_0	$\sigma(\beta_0)$	β_1	$\sigma(\beta_1)$	p-value
μ_m	1.0000	0.0253	0.0000	0.0076	1
μ_d	0.0000	0.0029	0.0016	0.0008	0.047
η	0.0517	0.0170	-0.0086	0.0038	0.030
p	0.5	0.0807	-	-	-
