# Supplementary material for: The Influence of Hydroxylation on Maintaining CpG Methylation Patterns: A Hidden Markov Model Approach
Source: PLoS Comput Biol. 2016 May 25;12(5):e1004905. doi: 10.1371/journal.pcbi.1004905 (PMC4880293; doi:10.1371/journal.pcbi.1004905)
Supplement: S4 Table — The p-values have been taken conducting a hypothesis test H0:β1λ=0∧β2λ=0 using the Wald statistic. (PDF) [file pcbi.1004905.s009.pdf]

DNA region	β_0^λ	β_1^λ	β_2^λ	p-value
IAP	0.9491	-0.0111	$6.05 \cdot 10^{-4}$	$< 10^{-5}$
L1mdA	0.8705	-0.0055	$1.40 \cdot 10^{-5}$	0.187
L1mdT	0.7378	-0.0011	$3.89 \cdot 10^{-5}$	0.005
mSat	0.8962	-0.0065	$1.21 \cdot 10^{-4}$	$< 10^{-5}$
MuERVL	0.8440	-0.0347	$1.69 \cdot 10^{-3}$	$< 10^{-5}$
Afp	0.8203	0.0059	$1.68 \cdot 10^{-5}$	$< 10^{-5}$
Ttc25	0.7440	-0.0435	$-2.95 \cdot 10^{-14}$	$< 10^{-5}$
Zim3	0.8530	-0.0965	$-1.16 \cdot 10^{-14}$	$< 10^{-5}$
Snrpn	1.0000	$-2.89 \cdot 10^{-11}$	$-4.44 \cdot 10^{-14}$	1.000
