# Supplementary material for: The Influence of Hydroxylation on Maintaining CpG Methylation Patterns: A Hidden Markov Model Approach
Source: PLoS Comput Biol. 2016 May 25;12(5):e1004905. doi: 10.1371/journal.pcbi.1004905 (PMC4880293; doi:10.1371/journal.pcbi.1004905)
Supplement: S5 Table — Computed Kullback-Leibler divergence and Bhattacharya distance values given by LOOCV data to compare the test error for assuming linear vs constant efficiencies. (PDF) [file pcbi.1004905.s010.pdf]

DNA region	KL-const	KL-linear	KL gain	BC-const	BC-linear	BC gain
IAP	0.164	0.131	20.1 %	5.33e-03	4.38e-03	17.8 %
L1mdA	0.026	0.023	11.5 %	8.10e-04	7.18e-04	11.4 %
L1mdT	0.101	0.099	1.9 %	3.18e-03	3.17e-03	0.3 %
mSat	0.163	0.162	0.6 %	5.09e-03	5.00e-03	1.8 %
MuERVL	0.497	0.321	35.4 %	1.62e-02	1.02e-02	37.0 %
Afp	0.149	0.114	23.5 %	4.79e-03	3.66e-03	23.6 %
Ttc25	0.209	0.171	18.2 %	7.03e-3	6.07e-3	13.7 %
Zim3	0.342	0.211	38.3 %	1.13e-2	7.00e-3	38.1 %
Snrpn	0.194	0.192	1 %	1.13e-2	7.00e-3	1 %
